# Genomic Insight into the Host–Endosymbiont Relationship of *Endozoicomonas montiporae* CL-33^T^ with its Coral Host

**DOI:** 10.3389/fmicb.2016.00251

**Published:** 2016-03-08

**Authors:** Jiun-Yan Ding, Jia-Ho Shiu, Wen-Ming Chen, Yin-Ru Chiang, Sen-Lin Tang

**Affiliations:** ^1^Biodiversity Research Center, Academia SinicaTaipei, Taiwan; ^2^Department of Seafood Science, Laboratory of Microbiology, National Kaohsiung Marine UniversityKaohsiung, Taiwan

**Keywords:** *Endozoicomonas*, host–bacteria interaction, comparative genomics, endosymbiosis, coral holobiont

## Abstract

The bacterial genus *Endozoicomonas* was commonly detected in healthy corals in many coral-associated bacteria studies in the past decade. Although, it is likely to be a core member of coral microbiota, little is known about its ecological roles. To decipher potential interactions between bacteria and their coral hosts, we sequenced and investigated the first culturable endozoicomonal bacterium from coral, the *E. montiporae* CL-33^T^. Its genome had potential sign of ongoing genome erosion and gene exchange with its host. Testosterone degradation and type III secretion system are commonly present in *Endozoicomonas* and may have roles to recognize and deliver effectors to their hosts. Moreover, genes of eukaryotic ephrin ligand B2 are present in its genome; presumably, this bacterium could move into coral cells via endocytosis after binding to coral's Eph receptors. In addition, 7,8-dihydro-8-oxoguanine triphosphatase and isocitrate lyase are possible type III secretion effectors that might help coral to prevent mitochondrial dysfunction and promote gluconeogenesis, especially under stress conditions. Based on all these findings, we inferred that *E. montiporae* was a facultative endosymbiont that can recognize, translocate, communicate and modulate its coral host.

## Introduction

The bacterial genus *Endozoicomonas* was first proposed by Kurahashi and Yokota (2007). All culturable species of this genus were isolated from marine invertebrates, including sea slug (Kurahashi and Yokota, 2007), encrusting coral (Yang et al., 2010), sponge (Nishijima et al., 2013), octocorals (Pike et al., 2013), and comb pen shell (Hyun et al., 2014). In culture-independent bacterial community studies, *Endozoicomonas* were frequently detected in various marine invertebrates and have been considered an important affiliate of the host holobiont, due to their positive association with healthy individuals, particularly in corals. For example, Vezzulli et al. (2013) compared bacterial communities on Mediterranean gorgonians (*Paramuricea clavata*) and reported that *Endozoicomonas* were a predominant group on healthy individuals, but declined greatly when their host was diseased. Similarly, Meyer et al. (2014) also noted that the *Endozoicomonas* was a predominant group in mucus of healthy corals, *Porites astreoides*, but was significantly less abundant when hosts were injured or diseased. Moreover, relative abundance of *Endozoicomonas* was altered following increased *p*CO_2_ in surrounding seawater or elevated seawater temperature (Morrow et al., 2015; Tout et al., 2015). Furthermore, the predominance of *Endozoicomonas* might also link to their host's prevalence in nature. In a survey of the distribution of the fungid coral *Ctenactis echinata*, Roder et al. (2015) reported that when *C. echinata* was most abundant, the microbiome was highly structured and dominated by *Endozoicomonas*. In contrast, when *C. echinata* was less abundant, bacterial communities became diverse and proportion of *Endozoicomonas* also decreased. A similar phenomenon was also reported by Roterman et al. (2015) regarding the oyster *Spondylus* in the Mediterranean Sea. In addition, two reports suggested that *Endozoicomonas* or *Endozoicomonas*-like bacteria might have a role in sulfur cycling (Raina et al., 2009) and production of antimicrobial compounds (Rua et al., 2014). Taken together, although numerous studies have highlighted the potential importance of *Endozoicomonas* for corals or reef invertebrates, the genetics, physiology, and ecology of these bacteria are mostly unknown.

*Endozoicomonas montiporae* CL-33^T^ was the first culturable coral-associated endozoicomonal bacteria, which was isolated from the encrusting pore coral *Montipora aequituberculata* at the southern coast offshore of Taiwan (Yang et al., 2010). That this species is culturable facilitates in-depth studies, particularly on genetics and physiology. Hence, in this study, we presented a high quality, nearly complete genome sequence of *E. montiporae*, and deciphered possible ecological functions of this bacterium, based on comparative genomic approaches and physiological tests. Our discoveries provided evidence-based insights regarding how *E. montiporae* interacted with its host from outside to inside of the coral cell. In addition, based on unique genomic features and newly discovered potential functions, we inferred that *E. montiporae* can be a facultative endosymbiont with the capability of helping its host to overcome environmental stresses.

## Materials and methods

### Bacterial strains, medium, and culture conditions

The strain of *E. montiporae* (CL-33^T^) was obtained from a previous study (Yang et al., 2010). Type strains of *Endozoicomonas elysicola* (DSM 22380^T^), *Endozoicomonas numazuensis* (DSM 25634^T^), and *Vibrio corallilyticus* (DSM 19607^T^) were purchased from Leibniz-Institut DSMZ—Deutsche Sammlung von Mikroorganismen und Zellkulturen GmbH (Braunschweig, Germany). Routine cultivation was done on MMB medium (per liter: 19.45 g of NaCl, 18.79 g of MgCl_2_·6H_2_O, 3.24 g of Na_2_SO_4_, 0.55 g of KCl, 0.16 g of NaHCO_3_, 0.12 g of CaCl_2_, 2 mg of KNO_3_, 8 mg of NaHPO_4_, 5 g of peptone, 1 g of yeast extract, 1 ml of trace element (Guillard and Ryther, 1962), 5.95 g of HEPES, pH 7.2), whereas for high-density cultivation of *E. montiporae*, 0.1% (w/v) of glucose/maltose was added to MMB. For sole carbon usage test, the MM medium (the same ingredients as MMB except the NaHPO_4_, peptone, and yeast extract were replaced by 100 μg of cyanocobalamin and 1 ml of 1M K-phosphate buffer) was used and test carbon sources, including glucose and testosterone, were added at 1% (w/v) and 500 μM, respectively. All bacterial strains were aerobically cultured at 25°C with 200 rpm agitation.

### Genomic DNA extraction, sequencing, and genome assemble

Genomic DNA of *E. montiporae* was extracted by the CTAB method, as described (Wilson, 2001). A physical map of *E. montiporae* genome was constructed from fresh cultures using an optical mapping technology, the ARGUS System (OpGen, Gaithersburg, ML, USA), at Yourgene Bioscience (New Taipei City, Taiwan). Purified genomic DNA was sequenced on three platforms: Illumina HiSeq 2000 (Illumina Inc., San Diego, CA, USA), Roche 454 GS Junior (Roche 454 Life Sciences, Branford, CT, USA) and Pacific Biosciences RS II (Pacific Biosciences, CA, USA). Three DNA libraries were prepared for Illumina sequencing: paired-end library (fragment size ~500 bp), PCR-free library (fragment size ~350 bp), and mate-paired library (fragment size ~4 Kbp). For pyrosequencing (454) and Pacific Biosciences (PacBio) sequencing, one flow cell and one SMRT cell were used for sequencing, respectively. The total read length from high-throughput sequencing was ~10,718 Mbp. Furthermore, CLC Workstation 6 (CLC bio, Aarhus, Denmark) was used to trim low-quality bases off Illumina and 454 raw reads. Illumina and PacBio reads were *de novo* assembled using ALLPATHS-LG (Butler et al., 2008) and SMRT-Analysis 2.2 (Pacific Biosciences, CA, USA), respectively. Scaffolds from Illumina and PacBio assemblies were analyzed in MapSolver™ (OpGen) to construct chromosomal scaffold by aligning the *Afl*II cutter (CTTAAG) identified in contig sequences to the optical map. Both GMcloser (Kosugi et al., 2015) and GapFiller (Nadalin et al., 2012) were used in gap filling with PacBio and Illumina reads, respectively. When using GMcloser, 454 reads were used to correct PacBio reads with the PBcR pipeline (Berlin et al., 2015) of Celera Assembler 8.2 (Myers et al., 2000) before gap filling. The sequences of rRNA operons were confirmed with molecular cloning method (see below).

### Genome annotation and bioinformatic analysis

The assembled genome was first subjected into non-coding gene predictions with tRNAscan-SE 1.3.1 (Lowe and Eddy, 1997), CRISPRFinder (Grissa et al., 2007), and using an HMM search with Rfam database 12 (Nawrocki et al., 2015). After masking predicted non-coding regions, coding genes were predicted with Prodigal-2.6 (Hyatt et al., 2010). Functional assignment for coding genes was accomplished with CDD (Marchler-Bauer et al., 2011), Pfam release 27 (Finn et al., 2014), COG 2014 update (Galperin et al., 2015), and NCBI PSI-BLAST against the Reference Sequence (RefSeq) database with the *e*-value threshold of 10^−6^. Metabolic pathways were analyzed with KEGG (Kanehisa and Goto, 2000) and MetaCyc (Karp et al., 2000). Gene annotations of the genome were manually revised and corrected using Artemis (Carver et al., 2011).

Prophage prediction was done with PHAST (Zhou et al., 2011). Repeat sequences were detected with UGENE (Okonechnikov et al., 2012), with a minimum length threshold of 500 bp and minimum identity of 98%. Regions containing rRNA genes (16S and 23S) were excluded from analyses. Repeat sequences were further identified using IS Finder database (https://www-is.biotoul.fr) with the *e*-value threshold of 10^−6^. Prokaryotic and eukaryotic subcellular location predictions were performed using Phobius (Käll et al., 2007) and BacelLo (set for prediction in animals; Pierleoni et al., 2006), respectively. The bacterial secretome was predicted using EffectiveT3 (set “gram-” for SignalIP, “type III effector prediction with animal set” for classification module and “selective” for cut-off; Arnold et al., 2009) and SSPred (set to use a Hybrid-II approach; Pundhir and Kumar, 2011). Coiled-coil prediction and homology modeling were performed using COILS (Lupas et al., 1991) and SWISS-MODEL (Biasini et al., 2014), respectively. GlycoEP (Chauhan et al., 2013) was used to predict *N*-glycosylation sites in eukaryotic proteins. A genome map was generated using Circos (Krzywinski et al., 2009). Visualization of protein models was done with UCSF Chimera v1.10.1 (Pettersen et al., 2004). Reference genomes/transcriptomes used in this study are listed in Table 1.

**Table 1:** **Reference genomes/transcriptomes used for comparative analysis in this study**.

**Organism name**	**Taxon (Phylum)**	**Lineage (Class/Order/Family)**	**References/Data source**
**ANIMAL**			
*Acropora digitifera*	Cnidaria	Anthozoa/Scleractinia/Acroporidae	Shinzato et al., 2011
*Acropora millepora*	Cnidaria	Anthozoa/Scleractinia/Acroporidae	Hemmrich and Bosch, 2008
*Aiptasia pallida*	Cnidaria	Anthozoa/Actiniaria/Actiniidae	Hemmrich and Bosch, 2008
*Anemonia viridis*	Cnidaria	Anthozoa/Actiniaria/Actiniidae	Hemmrich and Bosch, 2008
*Montastrea faveolata*	Cnidaria	Anthozoa/Scleractinia/Montastraeidae	Hemmrich and Bosch, 2008
*Nematostella vectensis*	Cnidaria	Anthozoa/Actiniaria/Edwardsiidae	Hemmrich and Bosch, 2008
*Porites astreoides*	Cnidaria	Anthozoa/Scleractinia/Poritidae	Hemmrich and Bosch, 2008
*Elysia chlorotica*	Mollusca	Gastropoda/-/Plakobranchidae	Bhattacharya et al., 2013
*Amphimedon quennslandica*	Porifera	Demospongiae/Haplosclerida/Niphatidae	Hemmrich and Bosch, 2008
*Leucosolenia complicata*	Porifera	Calcarea/Leucosolenida/Leucosoleniidae	Hemmrich and Bosch, 2008
*Oscarella carmela*	Porifera	Homoscleromorpha/Homosclerophorida/Plakinidae	Hemmrich and Bosch, 2008
*Sycon ciliatum*	Porifera	Calcarea/Leucosolenida/Sycettidae	Hemmrich and Bosch, 2008
**BACTERIA**			
*Alcanivorax borkumensis* SK2	*Proteobacteria*	*Gammaproteobacteria/Oceanospirillales/Alcanivoracaceae*	Schneiker et al., 2006
*Alcanivorax dieselolei* B5	*Proteobacteria*	*Gammaproteobacteria/Oceanospirillales/Alcanivoracaceae*	Lai et al., 2012
*Alcanivorax hongdengensis* A-11-3	*Proteobacteria*	*Gammaproteobacteria/Oceanospirillales/Alcanivoracaceae*	Lai and Shao, 2012
*Bermanella marisrubri* RED65	*Proteobacteria*	*Gammaproteobacteria/Oceanospirillales/Oceanospirillaceae*	Integrated microbial genomes
*Chromohalobacter israelensis* ATCC 43985	*Proteobacteria*	*Gammaproteobacteria/Oceanospirillales/Halomonadaceae*	Integrated microbial genomes
*Chromohalobacter salexigens* DSM 3043	*Proteobacteria*	*Gammaproteobacteria/Oceanospirillales/Halomonadaceae*	Integrated microbial genomes
*Endozoicomonas elysicola* DSM 22380	*Proteobacteria*	*Gammaproteobacteria/Oceanospirillales/Hahellaceae*	Neave et al., 2014
*Endozoicomonas numazuensis* DSM 25634	*Proteobacteria*	*Gammaproteobacteria/Oceanospirillales/Hahellaceae*	Neave et al., 2014
*Hahella chejuensis* KCTC 2396	*Proteobacteria*	*Gammaproteobacteria/Oceanospirillales/Hahellaceae*	Jeong et al., 2005
*Hahella ganghwensis* DSM 17046	*Proteobacteria*	*Gammaproteobacteria/Oceanospirillales/Hahellaceae*	Integrated microbial genomes
*Halomonas daqiaogenesis* CGMCC 1.9150	*Proteobacteria*	*Gammaproteobacteria/Oceanospirillales/Halomonadaceae*	Integrated microbial genomes
*Halomonas elongata* DSM 2581	*Proteobacteria*	*Gammaproteobacteria/Oceanospirillales/Halomonadaceae*	Schwibbert et al., 2011
*Halomonas halocynthiae* DSM 14573	*Proteobacteria*	*Gammaproteobacteria/Oceanospirillales/Halomonadaceae*	Integrated microbial genomes
*Kangiella aquimarina* DSM 16071	*Proteobacteria*	*Gammaproteobacteria/Oceanospirillales/Alcanivoracaceae*	Integrated microbial genomes
*Kangiella koreensis* DSM 16069	*Proteobacteria*	*Gammaproteobacteria/Oceanospirillales/Alcanivoracaceae*	Han et al., 2009
*Marinomonas mediterranea* MMB 1	*Proteobacteria*	*Gammaproteobacteria/Oceanospirillales/Oceanospirillaceae*	Integrated microbial genomes
*Marinomonas posidonica* IVIA-Po-181	*Proteobacteria*	*Gammaproteobacteria/Oceanospirillales/Oceanospirillaceae*	Integrated microbial genomes
*Thalassolituus oleivorans* MIL-1	*Proteobacteria*	*Gammaproteobacteria/Oceanospirillales/Oceanospirillaceae*	Integrated microbial genomes
*Vibrio coralliilyticus* ATCC BAA-450	*Proteobacteria*	*Gammaproteobacteria/Vibrionales/Vibrionaceae*	Kimes et al., 2012
*Vibrio harveyi* ATCC BAA-1116	*Proteobacteria*	*Gammaproteobacteria/Vibrionales/Vibrionaceae*	Integrated microbial genomes
*Vibrio shilonii* AK1	*Proteobacteria*	*Gammaproteobacteria/Vibrionales/Vibrionaceae*	Integrated microbial genomes

### Amplification, cloning, and sequencing *rrn* operons

Five operon-specific forward primers (rrnA_f, rrnB_f, rrnC_f, rrnD_f, and rrnE_f) and one universal reverse primer (rrnUniv_r; Supplementary Table S1) were designed from flanking regions of *rrnA*-*rrnE*. Target DNA fragments were amplified by using Phusion® High-Fidelity DNA Polymerase (New England BioLabs, Ipswich, MA, USA) and PCR reactants were prepared according to manufacturer's instruction. The amplification was done with 35 cycles of: 98°C for 10 s, 55°C for 30 s, 72°C for 190 s. Amplified products were separated with agarose gel electrophoresis and DNA bands with expected size (~5.5 Kbp) were eluted by QIAquick Gel Extraction Kit (Qiagen, Hilden, Germany). Purified DNAs were subjected into an A-tailing reaction with Ex Taq DNA Polymerase (TaKaRa, Shiga, Japan) and TA-cloning with pGEM-T vector system (Promega, Fitchburg, WI, USA) according to manufacturer's instruction. Cloned DNAs were transformed into One Shot® TOP10 Chemically Competent *E. coli* and transformants were screened with β-galactosidase α-complementation. Purification of plasmid DNAs and Sanger sequencing the insert DNAs were carried out at Genomics Inc. (New Taipei City, Taiwan). For complete sequencing the cloned rRNA operons, 16 universal walking primers were designed (Supplementary Table S1).

### Steroids degradation and detection

Test strains were aerobically cultured in MMB supplied with 0.1% (w/v) of maltose and 500 μM of testosterone. On each day, 1 ml of culture sample was taken, vigorously mixed with 0.8 ml of ethyl acetate and centrifuged (10,000 × *g*, 4°C, 10 min). After centrifugation, 0.6 ml of the upper-most layer was withdrawn and vacuum-dried. For detection of testosterone-derived compounds, dried samples were dissolved in 40 μl of ethyl acetate and 5 μl applied on TLC Silica gel 60 F_254_ plates (MERCK, Whitehouse Station, NJ, USA) and developed in dichloromethane:ethyl acetate:ethanol (7:2:0.5, v/v; Chiang et al., 2010). The separated metabolites were visualized by spraying on plates with 40% sulfuric acid solution, followed by heating at 150°C.

### Nucleotide sequence accession number

The genome sequence of *E. montiporae* and its annotation were deposited at GenBank under BioProject Number PRJNA66389 (Registration date: 28-Apr-2011).

## Results and discussion

### General genomic features

**Figure 1:**
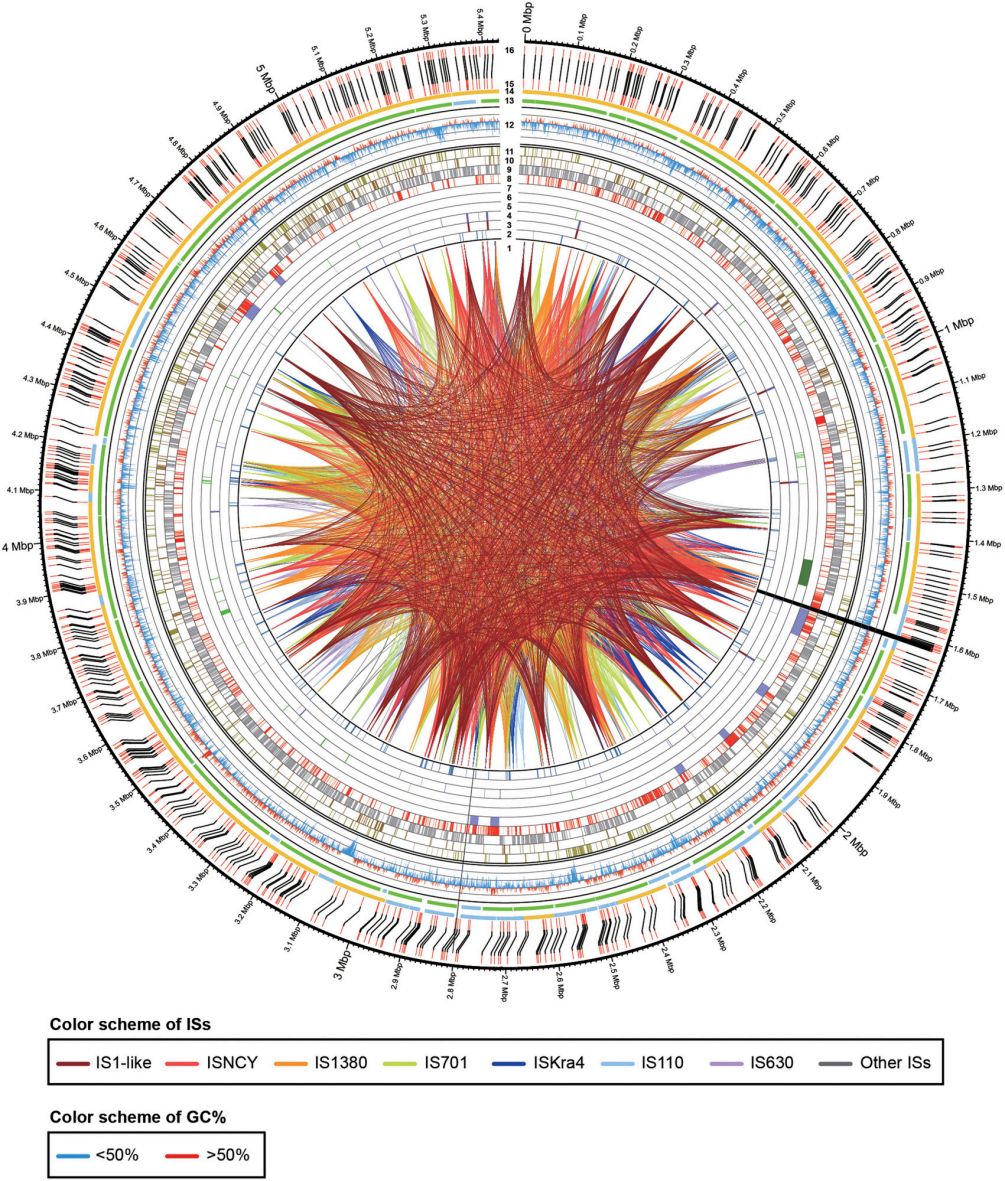
**Physical and genetic maps of the genome of ***E. montiporae*****. The track numbers represent: (1), IS linkages; (2), pseudogenes; (3), rRNA operons; (4), tRNAs; (5), eukaryotic domain proteins; (6), testosterone degrading gene cluster; (7), prophage regions; (8), unique genes in *E. montiporae*; (9), conserved genes in three *Endozoicomonas*; (10), genes shared with *E. numazuensis*; (11), genes shared with *E. elysicola*; (12), GC profile (red, > 50%; blue, < 50%); (13), ALLPATH-LG assembled scaffolds (green, orientated by MpSolver; light blue, orientated in gap filling process); (14), SMRTAnalysis assembled scaffolds (yellow, orientated by MpSolver; light blue, orientated in gap filling process); (15), *Afl*II pattern from assembled sequences; (16) physical map (*Afl*II cuts) generated with optical mapping technology. The black lines that connect track 15 and 16 indicated the alignment of *Afl*II cuts from assemble genome sequence and physical map. The two assembly gaps are indicated by black near position 1.6 and 2.8 Mbp.

The extracted genomic DNA of *E. montiporae* was sequenced though five libraries in three next-generation platforms and reads were assembled into one circular chromosome ~5.43 Mbp and ~1900-fold in coverage depth. Two gaps (~9.8 and 1.0 Kbp) remained (genome completeness was approximately 99.80%). At the beginning, lengths of assembled contigs from Illumina/454 reads were highly fragmented and *in silico Afl*II patterns of contigs were not properly matched to the physical map generated by optical mapping technology. We also noticed that a previously released *E. montiporae* genome (Neave et al., 2014) had the same limitation, which was attributed to sequence assemblies being greatly affected by numerous repeat sequences (which are longer than sequencing reads). Therefore, PacBio long-read sequencing technology was used. After incorporating long-reads, the final assembly had good agreement in *Afl*II cutting pattern (indicates the genome was accurately assembled; Figure 1). Detailed genome statistics are listed (Table 2).

**Table 2:** **Generic features of the ***E. montiporae*** draft genome sequence**.

**Parameter**	***E. montiporae***
Genome size (estimated from optical mapping)	5,432,010 bp
Total size of genome assembly	5,430,252 bp
GC content	48.37%
Number of coding sequences	4572
Number of rRNA operons	7
5S rRNA gene	8
16S rRNA gene	7
23S rRNA gene	7
Number of tRNAs	109
Number of non-coding genes	55
Number of pseudogenes/frame-shifted genes	198
Number of CPISPR arrays	5

The genome contained seven rRNA operons. The 16S-23S rRNA gene copy number in *E. montiporae* differed from that in other reported endozoicomonal genomes (16S/23S: *E. elysicola*, 6/6; *E. numazuensis*, 5/2; *E. montiporae*, 4/4; Neave et al., 2014). In contrast, the copy number of 5S rRNA gene was 8 (*E. elysicola* and *E. montiporae*, 8; *E. numazuensis*, 3; Neave et al., 2014). The rRNA operon *rrnD* was the only operon that contained two copies of 5S rRNA genes (Supplementary Information). In the orthology analysis, *E. montiporae* shared ~59.49 and ~63.09% coding genes with *E. elysiola* and *E. numazuensis* genomes, respectively. In the metabolic pathway analysis, the three species had the potential to synthesize all proteinogenic amino acids and many cofactors, prosthetic group and electron carriers required for growth, except vitamin B12.

**Figure 2:**
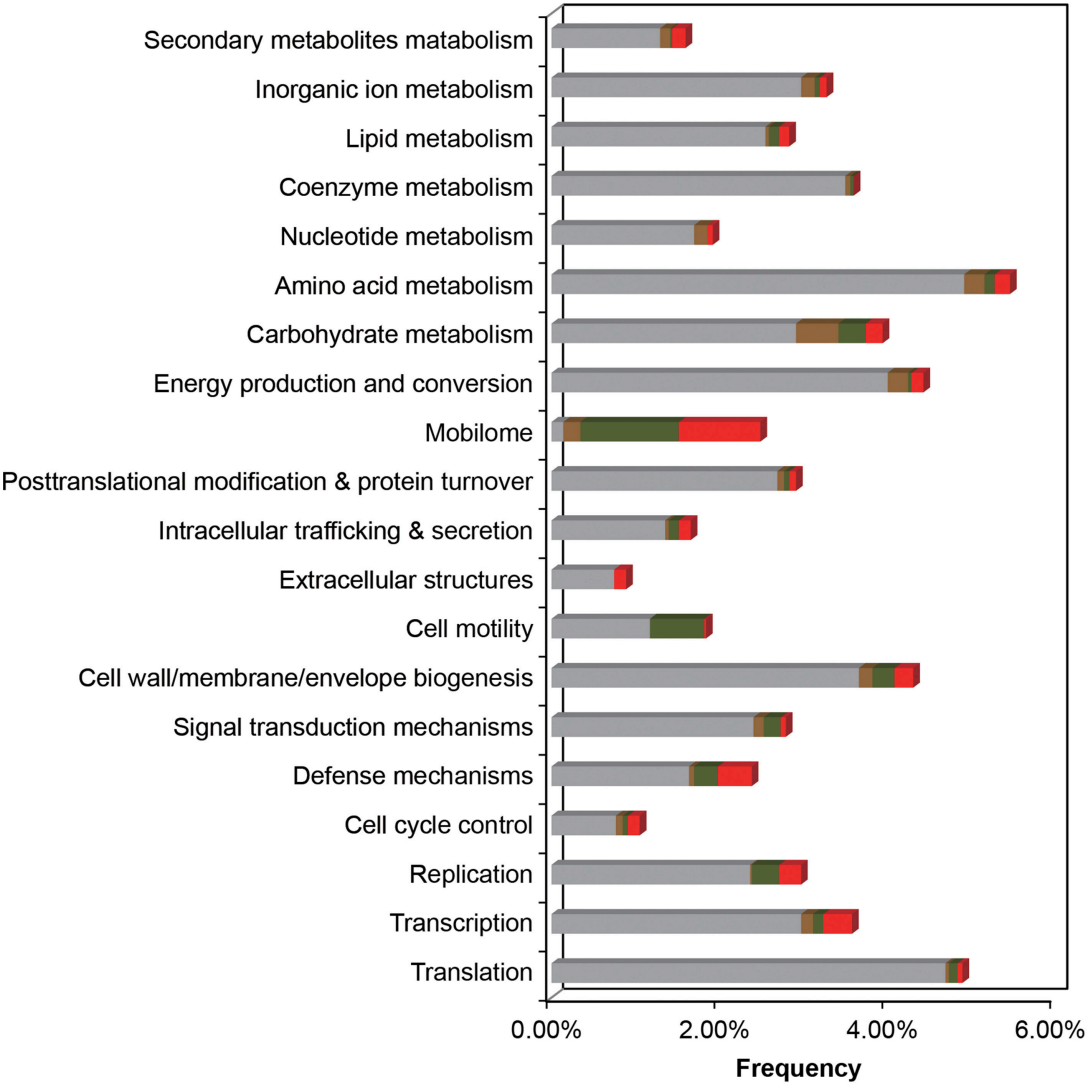
**Functional COG profile of the ***E. montiporae*** genome**. Genes shared in three *Endozoicomonas*, with *E. numazuensis*, with *E. elysicola*, or unique in *E. montiporae* are highlighted as gray, brown, green, and red with respectively. Corresponding locations are shown in Figure 1 (track 8–11).

In the comparative analysis, there were 1179 coding genes that could only be detected in *E. montiporae* (Figure 1). Based on the COG functional profile, *E. montiporae* had a higher proportion of genes (2.5% of all COGs) related to the mobilome (Figure 2). Many genes within the mobilome were similar to phage structural proteins (i.e., phage capsid or tail proteins), indicating frequent interactions between *E. montiporae* and its infectious agents, bacteriophages. Based on prophage prediction, there were eight prophages in the genome (ranging from 15.5 to 75 Kbp; Table 3). Four of those prophages were predicted as intact lysogenic phages, similar to *Vibrio* phage vB VpaM MAR (region 1 in the genome), *Vibrio* phage VPUSM 8 (regions 2 and 3), and *Escherichia* phage vB_EcoM-ep3 (region 4). In contrast, no prophage was detected in the draft genome of *E. elysicola* and *E. numazuensis*.

**Table 3:** **Prophage regions identified in the genome of ***E. montiporae*****.

**Region**	**Position**	**Length (Kbp)**	**Completeness**	**PHAST score**	**ORF number**	**Unique ORFs**	**Possible phage**
1	1624494–1699528	75	Intact	150	99	69	*Vibrio* phage vB VpaM MAR (NC_109722)
2	2025905–2046887	20.9	Intact	140	25	25	*Vibrio* phage VPUSM 8 (NC_022747)
3	2717260–2741388	24.1	Intact	140	32	31	*Vibrio* phage VPUSM 8 (NC_022747)
4	4621590–4662332	40.7	Intact	150	67	62	*Escherichia* phage vB_EcoM-ep3 (NC_025430)
5	1858945–1891753	32.8	Incomplete	60	31	13	*Staphylococcus prophage* phiPV83 (NC_002486)
6	2179474–2203283	23.8	Incomplete	40	21	10	*Stenotrophomonas* phage S1 (NC_011589)
7	2774561–2790090	15.5	Incomplete	50	28	24	*Pseudoalteromonas* phage PM2 (NC_000867)
8	4748489–4767285	18.7	Incomplete	30	27	21	*Rhizobium* phage RR1-B (NC_021557)

The *E. montiporae* genome contained at least 452 mobile elements, which include 46 IS1-like IS elements (most active), 36 of Group II introns and 370 of integrase/transposase coding genes from various IS families. The estimated repeat coverage in *E. montiporae* was ~9.07%, greatly higher than *E. elysicola* and *E. numazuensis*, which were only ~0.11 and ~4.05%, respectively (Figure 1, also see Supplementary Figure S1). Those mobile elements were likely still active in *E. montiporae*, because 56 genes were inactivated due to insertions (Supplementary Table S2).

### *Endozoicomonas montiporae* is a testosterone degrader

**Figure 3:**
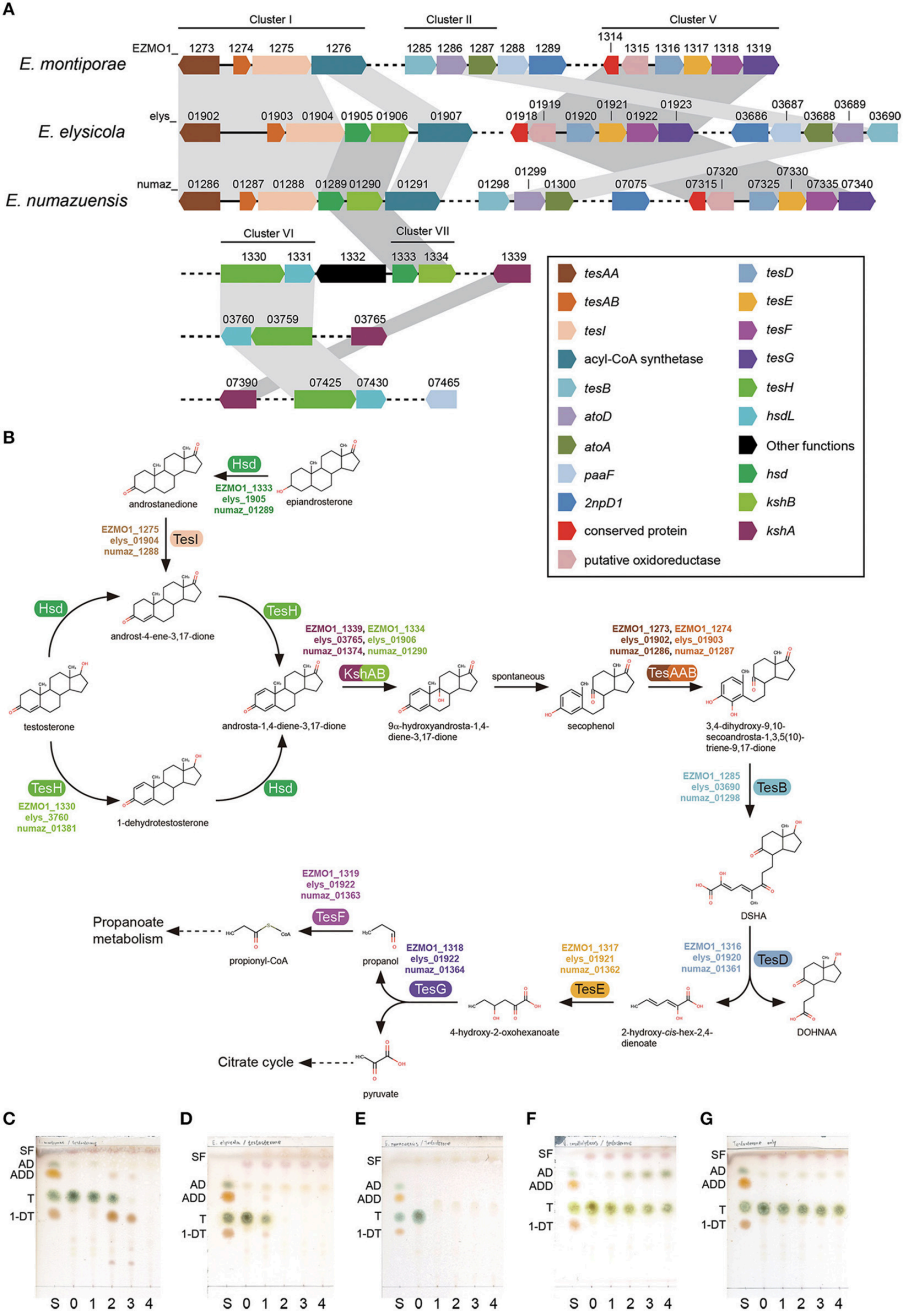
**Testosterone degradation in ***Endozoicomonas*****. The gene order of key genes required for degradation **(A)** and their corresponding reactions **(B)** presented in *Endozoicomonas* species. Degrading capabilities of test bacteria were confirmed by supplying 500 μM of testosterone in culture medium and sampled daily in the previous culture. Test bacteria are: *E. montiporae* **(C)**, *E. elysicola* **(D)**, *E. numazuensis* **(E)**, *V. corallilyticus* **(F)**, and a blank control without inoculum **(G)**. Abbreviations used in TLC analysis are: 1-DT, 1-dehydrotestosterone; AD, androst-4-ene-3,17-dione; ADD, androsta-1,4-diene-3,17-dione; S, standard mixture; SF, solvent front; T, testosterone. Numbers stand for the day of culture.

The three endozoicomonal species in the present study shared high similarities in metabolic capacities. It was noteworthy that they all had genes for degrading testosterone (male sex hormone), although that was rarely reported in oceanospirial genomes. Key genes required for degrading testosterone were detected within five of seven conserved gene clusters (I, II, V, VI, and VII) in the endozoicomonal genomes (Figure 3A, Supplementary Table S3). From a reconstructed pathway, testosterone could be degraded into propionyl-CoA and pyruvate through the 9,10-seco pathway (Coulter and Talalay, 1968). Furthermore, the first two metabolites could be further utilized (via propionate metabolism and TCA cycle) in the *Endozoicomonas* species (Figure 3B). Clusters III and IV were mainly composed of genes related to fatty acid β-oxidation (Supplementary Table S3). Interestingly, those genes formed conserved synteny with known aerobic testosterone-degrading bacteria (Horinouchi et al., 2012), indicating those genes were probably acquired via horizontal gene transfer. In our degradation tests, all three *Endozoicomonas* were able to completely degrade testosterone (Figures 3C–E, with *Endozoicomonas*; Figure 3G, medium only control). In contrast, there were no genes related to testosterone degradation identified in *Vibrio* species and the coral pathogen *V. corallilyticus* had a negative reaction regarding testosterone degradation (Figure 3F). When minimal medium was used to determine whether testosterone could serve as sole carbon source for growth, only *E. elysicola* and *E. numazuensis*, but not *E. montiporae*, grew (albeit with a very low biomass; i.e., OD_600_ ~0.1). Low testosterone concentrations can be detected in coral reef seawater, with highest concentrations in coral tissues (42.7 ng/g tissue in conjugated form; Twan et al., 2006). However, the concentration was still ~5,300-fold lower than our tests, suggesting that testosterone might not be a nutrient but more like an “animal cue” for *Endozoicomonas*.

### *Endozoicomonas montiporae* possesses a *N*-deglycosylation enzyme that might help it to penetrate host's mucus layer

**Figure 4:**
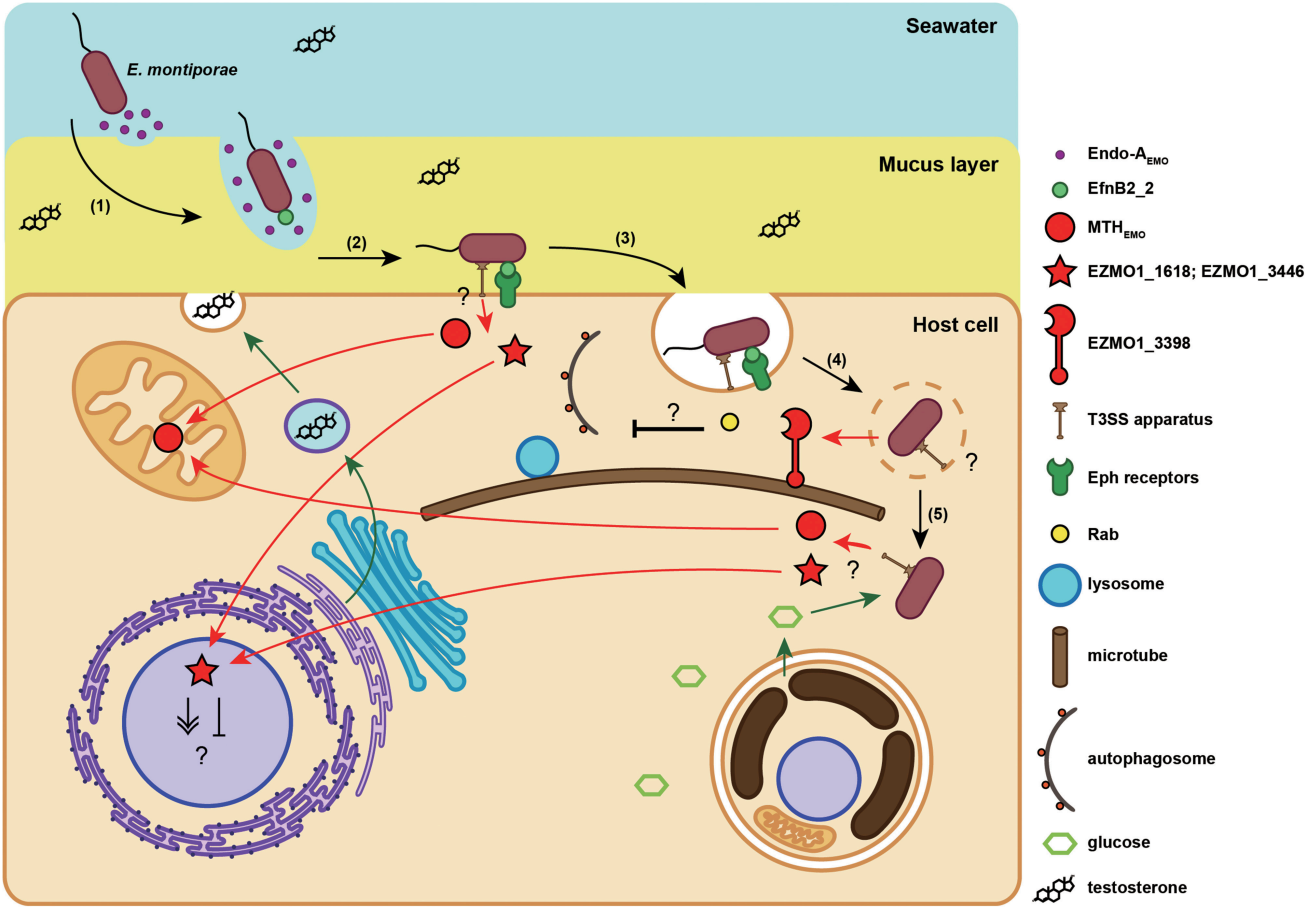
**Proposed infection model of the ***E. montiporae*****. When *E. montiporae* attaches to the host mucus layer, secreted Endo-A_EMO_ could reduce mucus viscosity, enabling the bacterium to penetrate through the mucus (1). Once the bacterium reached the host plasma membrane, the EfnB2_2 could bind with host Eph receptors (2), and initiate endocytosis (3). When *E. montiporae* enters into a host cell, it might use extracellular enzymes to disrupt the endosome and interfere with phagolysosome maturation by chelating Rabs with EZMO1_3398 (4). After escaping the endosome, *E. montiporae* could utilize nutrients in the host cytoplasm, particularly glucose produced by endosymbiotic zooxanthellae (5).

EZMO1_4380 (*endo-A*_Emo_) was a unique gene in *E. montiporae*. Its translated product was similar to endo-β-*N*-acetylglucosaminidase A (PDB number: 2VTF) from *Arthrobacter protophormiae* (Supplementary Figure S2). Endo-β-*N*-acetylglucosaminidases (ENGases) have been reported in a wide variety of organisms, with roles in hydrolyzing glycosidic bond present in *N*-linked sugar chains in glycoproteins (Yin et al., 2009). In contrast to those cytoplasmic ENGases in plants and animals, bacterial and fungal ENGases are secreted enzymes (Suzuki et al., 2002). The Endo-A_Emo_ contained a signal peptide sequence at its N-terminus (1–25 residues), a hallmark feature of a secreted enzyme. The predicted function of this protein might be related to digest glycoproteins, which were commonly present in the natural habitats of *E. montiporae*, e.g., coral mucus. Glycoprotein mucins are the major structural component of coral mucus. However, only the *O*-linkage was experimentally detected in coral mucins (Meikle et al., 1987). In genomic surveys, the starlet sea anemone *Nematostella vectensis* has an *N*-glycosylated mucin coding gene (XP_001636262; Lang et al., 2007). In addition, in the draft genome of the coral *Acropora digitifera*, open reading frames were detected in two regions located inside the scaffold 125 and 132 that resembled the mucin protein of *N. vectensis* and contained putative *N*-glycosylation sites. Therefore, we inferred that coral mucins could be *N*-glycosylated and physical characteristics might be affected due to deglycosylation by Endo-A_Emo_. Blocking *N*-glycosylation in rat Muc2 reduced gel formation and/or mucin viscoelasticity (Bell et al., 2003). Hence, relaxation of mucin by Endo-A_Emo_ would facilitate *E. montiporae* to pass through coral mucus. Actually bacteria degrade mucin by two types of reactions, namely *O*-linkage hydrolysis and proteolysis (McGuckin et al., 2011). However, no similar homologs related to enzymes of the two reactions were detected in *Endozoicomonas* genomes, suggesting the bacteria might not be able to deeply decompose their host mucus. Perhaps the role of Endo-A_Emo_ was not solely related to mucus dissociation, but it could help the bacterium to reach to specific host receptors on the cell membrane (Figure 4), similar to other bacteria (McGuckin et al., 2011).

### Host's ephrin receptors might be involved in endocytosis of *E. montiporae*

Two genes, EZMO1_1051 (*efnB2_1*) and EZMO1_3739 (*efnB2_2*), encoded proteins similar to eukaryotic proteins, but lacking orthologs in other bacteria. In that regard, EfnB2_1 and EfnB2_2 corresponded to NEMVEDRAFT_v1g212995 and NEMVEDRAFT_v1g236436 from *N. vectensis*, with 39 and 34% amino acid identities, respectively. Both EfnB2_1 and EfnB2_2 contained ephrin ectodomain (cd02675) and secretion signals at their N-terminus. Besides, EfnB2_2 encompassed transmembrane regions and was predicted as a membrane protein that could adhere on a cell surface, whereas EfnB2_1 was not. Tertiary structures of the two proteins, predicted by homology modeling, were very similar to each other, and they also resembled type B ephrin-B2 from mouse (1IKO; Supplementary Figure S3). However, it is noteworthy that the ephrin ligand protein was similar to the cupredoxin domain, a protein domain containing copper binding sites and usually responsible for electron-transfer reactions in bacteria (De Rienzo et al., 2004). Distinct from cupredox domain proteins, ephrin acts as a signal molecule in animals, with no metal-binding capability (Toth et al., 2001). For the two proteins, EfnB2_1 and EfnB2_2, all lacked copper binding residues by aligning with bacterial cupredoxins (azurins and auracyanins), indicating that the functions of EfnB2_1 and EfnB2_2 were more similar to eukaryotic ephrin ligand.

In vertebrates, ephrins are ligands for ephrin receptors (Ephs) and binding can activate intracellular signal pathways involved in a variety of cellular functions, including differentiation, migration, segmentation, and endocytosis (Kullander and Klein, 2002; Pitulescu and Adams, 2010). In cnidarian, ephrins, and Ephs in *Hydra vulgaris* belonged to type B (Tischer et al., 2013). Furthermore, ephrin ligands and receptors were strongly expressed on endoderm of tentacles and buds, suggesting ephrin-Eph signaling might have roles in cell adhesion and tissue boundary formation in *H. vularis* (Tischer et al., 2013). Ephrin-Eph signaling in corals has never been discussed; however, ephrin/Eph receptor genes were present in the genome of *A. digitifera* and the ephrin ligand gene was differentially expressed in *A. palmata* larvae (Polato et al., 2013), indicating that ephrin-Eph signaling could have roles in corals.

Although, the reason why *E. montiporae* has ephrin genes remains unknown, the rare occurrence of eukaryotic cell surface ligand coding genes might have a role in targeting host receptor and initiating internalization. In contrast, pathogenic bacteria can use azurin, a cupredoxin protein similar to ephrin in structure, to invade host cells. For example, bacterial azurin can solely enter the murine macrophage J774, as well as several types of cancer cells and trigger programed cell death of mammalian cell lines by interfering with Eph signal pathways (Yamada et al., 2005; Chaudhari et al., 2007). Furthermore, azurin in the human pathogen *Neisseria gonorrhoeae* is essential for survival inside host cells (Wu et al., 2005). Azurin othologous genes were only detected in three pathogenic *Vibrio* genomes, but not in the three *Endozoicomonas*. Accordingly, we speculated that the ephrin ligand in *E. montiporae* could serve as a “cloak” and facilitate this bacterium to enter host cells, but would not impair its host (Figure 4).

### A giant secreted protein of *E. montiporae* might modulate host's intracellular vesicle trafficking

To survive inside a host cell, intracellular pathogens and symbionts have to evade the host defense system, which relies on intracellular vesicle trafficking (Davy et al., 2012; Ashida et al., 2015). In the present study, it was determined that *E. montiporae* contained at least one gene that may be essential for this purpose. In that regard, EZMO1_3398 is a giant extracellular protein-coding gene, which encodes 3872 amino acids and comprises the myosin tail (pfam01576) and *Mycoplasma* helix-rich (TIGR04523) domains at its N-terminus (Supplementary Figure S4A). Furthermore, EZMO1_3398 was predicted as a Sec-dependent secreted protein but not a membrane protein. The myosin tail domain contains α-helices that can form a coiled-coil structure for dimerization (Krendel and Mooseker, 2005). Regarding the *Mycoplasma* helix-rich domain, it is still functionally unclear, although it is common in *Mycoplasma* species.

Based on structure prediction, EZMO1_3398 could be a chimeric protein, encompassing bacterial and eukaryotic protein features. Potential functions could involve modulating host's intracellular trafficking processes, particularly during initial stages of infection (Figure 4). Three protein substructures were detected in the EZMO1_3398, including the tail of myosin (1I84), LidA (3TNF), and stalk with microtubule-binding domain from dynein 2 (4RH7), at its first half (Supplementary Figure S4A). The LidA-like structure was a core domain that connected the myosin tail-like and the dynein 2 stalk-like structures to its helix-1 and helix-7, respectively (Figures S4C–E). However, no myosin/dynein motor domains were identified, neither in sequence nor in structure similarities, in EZMO1_3398. The LidA protein of *Legionella penumophila* is a Rab supereffector protein that strongly binds to host's Rab proteins and might help *L. penumophila* to survive inside a host cell by preventing autophagosome maturation (Joshi and Swanson, 2011; Schoebel et al., 2011). Interfering with Rab proteins inside hosts seems to be a common strategy also used by endosymbionts to establish a symbiotic relationship. Even though a detailed mechanism is unclear, study of *Symbiodinium-Aiptasia pulchella* symbiosis suggests the symbiotic zooxanthellae could manipulate endosomal trafficking and prevent lysosome fusion (Davy et al., 2012). Rab proteins can perform several cellular functions when binding to various effector proteins. Regarding vesicle movement, Rabs can recruit myosins or dyneins to exert movement along microtubes. Binding with effectors and GTPase activating protein can activate the GTPase activity of Rab and hydrolysis of GTP can trigger movement (Hutagalung and Novick, 2011). Interestingly, EZMO1_3398 contained substructures similar to myosin and dynein and both are connected with the Rab-binding central domain. Therefore, the EZMO1_3398 might be able to attach host's microtubes via binding certain Rab proteins for some functional purposes, e.g., to prevent lysosome fusion (Figure 4).

### Type III secretion effector proteins might help *E. montiporae* to infect coral host and mediate host's metabolism

A common feature of the *Endozoicomonas* species in this study is that they all had a host-dependent secretion system, the type III secretion system (T3SS), a well-known bacterial secretion system that is functionally involved in bacteria–host interactions, either symbiosis or pathogenesis (Preston, 2007). The presence of T3SS in the *Endozoicomonas* suggests they are capable of interacting with their hosts. To identify potential interactions between *E. montiporae* and its coral host, we analyzed predicted T3SS secretomes and the possible subcellular locations of those secreted proteins (i.e., effectors). There were 242 proteins predicted to be T3SS effectors, including many hypothetical proteins (Supplementary Table S4). Among those possible T3SS effectors, five proteins might be involved in survival inside hosts, regulating host's metabolism and/or increasing host's fitness.

EZMO1_0953 is a catalase gene of *E. montiporae* and its product was one of the T3SS effectors. Besides, catalase was a common T3SS-screted protein in other *Endozoicomonas*. Secretion of catalase by a host-dependent system indicated that the H_2_O_2_-scavenging enzyme might be crucial for *Endozoicomonas* to live within their hosts. For animal-associated bacteria, including pathogens, parasites, or symbionts, catalase activity is important for them to survive in their hosts (Bishai et al., 1994; Rocha et al., 1996; Visick and Ruby, 1998). Interestingly, instead of catalase, we identified another type of H_2_O_2_-removing enzyme, the thiol peroxidases (Tpx), that could be secreted via T3SS in pathogenic bacteria *Vibrio*. Tpx is a part of T3SS assembly in the human pathogen *Yersinia* species and also connects to effector delivery (Wang et al., 2011). In Shiga toxin-producing *Escherichia coli* O157:H7, Tpx is required for biofilm formation on the human HT-29 cell line (Kim et al., 2006). The same gene was detected and predicted as a T3SS-secreted protein in our analysis. Collectively, we speculated that the T3SS-secreted antioxidant profile could be a distinguishable signature between pathogenic and non-pathogenic bacteria.

EZMO1_3421 encodes isocitrate lyase (ICL_Emo_), a key enzyme of the glyoxylate cycle, and was a predicted T3SS secreted protein in *E. montiporae*. The glyoxylate cycle is common in bacteria, fungi and plants, and can bypass carbon flow from fatty acid degradation to gluconeogenesis. Glyoxylate cycle enzymes are also present in nematodes and cnidarians (Kondrashov et al., 2006) and expression of ICL was detected in transcriptomes of *A. millepora* larvae and *A. palmate* (Meyer et al., 2009; Polato et al., 2013).

**Figure 5:**
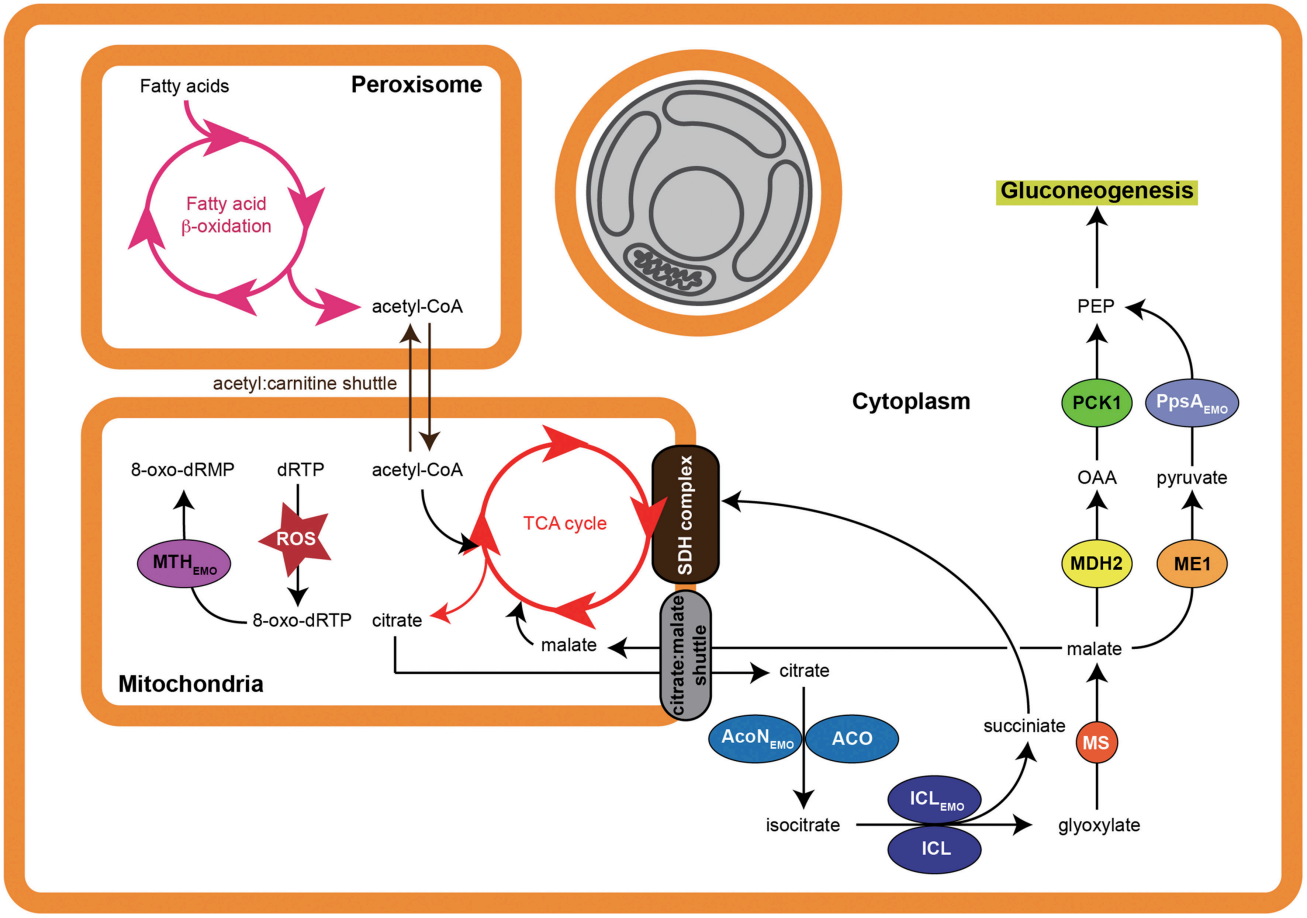
**Proposed pathway modulation model of ***E. montiporae*****. When the coral host is under stress, loss of symbiotic zooxanthellae (indicated by gray) makes host cell starts to use fatty acids and convert them into glucose. Active energy production and metabolite exchange could lead to accumulation of reactive oxygen species (ROS) in mitochondria matrix, which can oxidize deoxyribose purine triphosphates (dRTP) into 8-oxo-dRTP and cause mutation. The T3SS secreted enzymes could hasten carbon flow from fatty acids to glucose and hydrolyze 8-oxo-dRTP inside mitochondria. The abbreviations of host enzymes are: ACO, aconitate hydratase; MS, malate synthase; ME1, NADP-dependent malic enzyme; MDH2, malate dehydrogenase (cytoplasmic).

In addition to ICL_Emo_, there were two other T3SS effectors in *E. montiporae* that could concurrently work with the host enzymes for gluconeogenesis, namely EZMO1_2385 and EZMO1_2390 that encode the aconitate hydratase (AcoN_Emo_) and phosphoenolpyruvate synthase (PpsA_Emo_), respectively. In bacteria, AcoN and PpsA can redirect metabolites from the TCA cycle to gluconeogenesis. Up-regulation of fatty acid β-oxidation, glyoxylate cycle and gluconeogenesis have been proposed to enable corals to convert stored fatty acids to carbohydrates for surviving stress-induced starvation (Kenkel et al., 2013). Hence, coupling of T3SS secreted ICL_Emo_, AcoN_Emo_, and PpsA_Emo_ from *E. montiporae* might increase metabolic efficiency of gluconeogenesis in the coral host. Furthermore, such metabolism integration might promote host survival under stressful conditions (Figure 5).

Interestingly, we identified ICL orthologs that did not belong to T3SS effectors in *E. elysicola* and *E. numazuensis*. Notably, glyoxylate cycle enzymes were not detected in the representative genomes for their hosts (i.e., sea slug and sponge; Table 4). Besides, the predicted T3SS secretomes of *Endozoicomonas* were less common (Supplementary Table S4). Therefore, *Endozoicomonas* might have evolved unique strategies to interact with their own specific hosts.

**Table 4:** **Glyoxylate cycle enzymes (ICL and MS) in selected marine invertebrates**.

**Type**	**Organism name**	**Data source**	**Number of BLAST hit**	
			**ICL**	**MS**
Coral	*Acropora digitifera*	Genome	1	2
	*Acropora millepora*	ESTs	1	2
	*Montastrea faveolata*	ESTs	3	1
	*Porites astreoides*	ESTs	2	2
Sea anemone	*Aiptasia pallida*	ESTs	1	2
	*Anemonia viridis*	ESTs	1	0
	*Nematostella vectensis*	Genome	1	2
Sea slug	*Elysia chlorotica*	SRA	0	0
Sponge	*Amphimedon quennslandica*	Genome	0	0
	*Leucosolenia complicata*	Genome	11	2
	*Oscarella carmela*	Genome	0	0
	*Sycon ciliatum*	Genome	2	0

*1. ICL and MS sequences from Saccharomyces cerevisiae were used as representitive sequences (queries)*.

*2. When searching in EST database, tblastn was used and thresholds were set: e-value < 1e-15; bitscore > 100*.

*3. When searching in SRA database, tblastn was used first. Reads may affiliate with query sequences were recovered and subjected into blastn search against to nr/nt database. In blastn search, reads shared high identities and coverages to bacterial subjects will be excluded*.

The EZMO1_3450 (*mth*_Emo_) encodes the 7,8-dihydro-8-oxoguanine triphosphatase (MTH) and was another T3SS effector. The predicted subcellular location in host cell of MTH_Emo_ was in the mitochondria. The MTH enzyme can hydrolyze damaged purine nucleoside triphosphates caused by attacks from reactive oxygen species and therefore can confer protection to the cell against various oxidative stresses. In animal models, MTH can prevent mitochondrial dysfunction, attenuate stress-induced cell death and enhance vitality (Ichikawa et al., 2008; De Luca et al., 2013). Even though the detailed mechanism is still unknown, bacterial endosymbiosis can prevent mitochondrial dysfunction in a mycorrhizal host and improve fitness of its fungal host during the pre-symbiotic rhizospheric phases (Salvioli et al., 2015). Exporting MTH_Emo_ from *E. montiporae* to the host's mitochondria might promote mitochondrial functions. In addition, because mitochondria are also involved in fatty acid oxidation, MTH_Emo_ could promote conversion of fatty acids to glucose in coral cells under bleaching stress (Figure 5). Accordingly, *E. montiporae* might be able to enhance host's fitness to environment changes by protecting mitochondria from the oxidative injury and/or altering carbon flows of host metabolism.

### T3SS effectors might also involve in modulating host's signaling pathways

Two eukaryotic signal pathway proteins, serine/threonine protein kinase (STPK; EZMO1_1618 and EZMO1_3446), were predicted as T3SS effectors in *E. montiporae*. Both EZMO1_1618 and EZMO1_3446 were similar to STPK genes from the protozoa *Trypanosoma congolense* (TCIL3000_11_7730; identities: 33%) and the fungus *Phytophthora nicotianae* (L916_19948; identities: 34%), respectively. The two proteins were unlikely to be membrane-bound receptors, due to a lack of transmembrane regions in their amino acid sequences. Furthermore, predicted subcellular locations of EZMO1_1618 and EZMO1_3446 were all in the host nucleus (Figure 4). Bacterial types of STPKs can have roles during infection of their hosts (Whitmore and Lamont, 2012; Canova and Molle, 2014). For example, the human pathogen *Yersinia* species can secrete STPK protein (i.e., YopO) into host cells via T3SS and can interfere with the actin cytoskeleton of macrophage to prevent phagocytosis (Black et al., 2000; Juris et al., 2000; Grosdent et al., 2002). In contrast, the T3SS-secreted STPKs in *E. montiporae* were not similar to bacterial counterparts regarding protein orthology and predicted subcellular locations. Perhaps the two STPKs in *E. montiporae* were involved in altering certain gene expression in their host's nucleus.

### *Endozoicomonas montiporae* could be a facultative coral endosymbiont

Based on niche specificity, special genomic characteristics, and growth features, we inferred that *E. montiporae* could be a facultative endosymbiotic bacterium in corals.

One of the indirect albeit rational evidences for niche specificity is that *Endozoicomonas* were mostly detected or isolated from tissues of marine invertebrates in reefs (Kurahashi and Yokota, 2007; Yang et al., 2010; Bourne et al., 2013; Nishijima et al., 2013; Pike et al., 2013; Hyun et al., 2014), implying their niches were likely associated with reef marine invertebrates. Moreover, the *Endozoicomonas* strains in this study all had genes of the host-dependent secretion system, carrying several putative animal genes and were able to degrade testosterone, strongly suggesting that the natural niche of those bacteria should be spatially close to or indeed within their animal hosts. We further deduced that the bacterial niches being intimate with host cells might be an important factor for gene transfer from hosts to bacteria.

Highly represented repeat sequences and pseudogenes in a bacterial genome could be a sign of genome erosion, a common feature in host-restricted symbionts or pathogens (McCutcheon and Moran, 2011). Occurrence frequencies of the IS and pseudogenes among the three endozoicomonal genomes could be grouped into various stages of genome erosion: *E. elysicola*, free-living; *E. numazuensis*, intermediate between free-living and host-restricted symbiont; and *E. montiporae*, a recently host-restricted symbiont/pathogen (Supplementary Figure S1) according to the category proposed by McCutcheon and Moran (2011). Such genomic features have been reported from an *E. elysicola*-like species, which may cause epitheliocystis in sharpsnout seabream larva (Katharios et al., 2015). Even though the genetic basis which can cause fish diseases remains unclear, increasing genome plasticity by accumulating a high proportion of insertion sequences or improving fitness to hosts by eliminating redundant genes through a pseudogenization process seemed to be a common strategy to survive inside host tissue. Furthermore, this was consistent with an evolutionary change toward tightly host-associated lifestyles that could occur in certain *Endozoicomonas* species.

Ecological roles of bacteria for mutualism or parasitism are commonly dynamic. Based on genomic analysis, we inferred that *E. montiporae* could be considered as a bacterium beneficial to corals. Not only does this bacterium have genes that could help its hosts, it was noteworthy that no known bacterial toxin homologs were present in its genome. However, potential parasitic roles of other *Endozoicomonas* species could not be excluded. For example, there are two reports that *E. elysicola*-like species could be associated with epitheliocystis in fish larvae (Mendoza et al., 2013; Katharios et al., 2015). However, these observations were different from previous surveys of marine invertebrates. Due to limited knowledge, it is unknown whether such different life styles of *Endozoicomonas* are due to genetic variations (e.g., presence or absence of virulence genes), physiological variations of their hosts, or both.

Some enzymes of *E. montiporae* could be secreted into host cytoplasm and promote glucose production in host cells. Glucose could be a critical factor in host cells and a feedback from the host cell, which can support growth of *E. montiporae*. In the present study, compared to other strains, *E. montiporae* did not achieve high-density growth in rich medium without glucose (Supplementary Figure S5). Therefore, supplying glucose could be a key to maintain a viable *E. montiporae* population inside corals.

To survive inside corals, it would be necessary for *E. montiporae* to communicate with its coral hosts as well as with endosymbiotic zooxanthellae, a key symbiotic partner inside coral cells. Glucose supply might be essential to maintain a symbiotic relationship between *E. montiporae* and coral. Therefore, it is also important to detect interactions between bacterial and algal endosymbionts. Therefore, we searched for potential proteins in the bacterium that could be involved in interactions, although none wa*s* detected. Unfortunately, our bioinformatics analyses lacked suitable models for prediction. In that regard, current prediction models of subcellular locations have not been established from corals or animals with algal endosymbionts. Therefore, for example, a protein could be translocated to specific organelles or compartments in which zooxanthellae reside, i.e., the symbiosome (Trench, 1979; Birkeland, 1997). It was noteworthy that eukaryotic domain protein coding genes present in *E. montiporae*, especially STPKs, were similar to proteins in invertebrates or fungi, but not plants. Therefore, we concluded that exchanges of genetic material between *E. montiporae* and zooxanthellae was unlikely. Although, our current results suggested the two endosymbionts might have no direct contact, we cannot assume that the *E. montiporae* does not communicate with algal symbionts, for example, by metabolites (Glick, 2012).

In conclusion, bacterial consortiums within corals are extremely complex and there has been a lack of detailed knowledge regarding interactions between bacteria and their coral host. Therefore, studying interactions between one bacterium and one coral species may provide more direct evidence and details. In this research, we focused on the *Endozoicomonas*, a group of bacteria present mainly on healthy coral, and how they may assist their hosts. We provided an abundance of genomic information, and discussed how *Endozoicomonas*, particularly *E. montiporae* could interact with their hosts. Our findings provided a valuable guide for future in-depth molecular or physiological studies for coral microbiology.

## Author contributions

ST, WC, and JS conceived of the work. JD and JS conducted whole genome sequencing and were involved in genome assemble. JC contributed to comparative genome analysis, functional gene prediction, and other bioinformatics analysis. YC and JD designed the experiment of testosterone degradation and was carried out by JD. JD and ST contributed to writing the manuscript and JD elaborated the figures and tables. All authors critically reviewed, revised and ultimately approved this final version.

### Conflict of interest statement

The authors declare that the research was conducted in the absence of any commercial or financial relationships that could be construed as a potential conflict of interest.

## References

[B1] Arnold R. Brandmaier S. Kleine F. Tischler P. Heinz E. Behrens S.. (2009). Sequence-based prediction of type III secreted proteins. PLoS Pathog. 5:e1000376. 10.1371/journal.ppat.1000376 19390696 PMC2669295

[B2] Ashida H. Mimuro H. Sasakawa C. (2015). *Shigella* manipulates host immune responses by delivering effector proteins with specific roles. Front. Immunol. 6:219. 10.3389/fimmu.2015.00219 25999954 PMC4423471

[B3] Bell S. L. Xu G. Khatri I. A. Wang R. Rahman S. Forstner J. F. (2003). N-linked oligosaccharides play a role in disulphide-dependent dimerization of intestinal mucin Muc2. Biochem. J. 373, 893–900. 10.1042/BJ20030096 12744721 PMC1223556

[B4] Berlin K. Koren S. Chin C.-S. Drake J. P. Landolin J. M. Phillippy A. M. (2015). Assembling large genomes with single-molecule sequencing and locality-sensitive hashing. Nat. Biotechnol. 33, 623–630. 10.1038/nbt.3238 26006009

[B5] Bhattacharya D. Pelletreau K. N. Price D. C. Sarver K. E. Rumpho M. E. (2013). Genome analysis of *Elysia chlorotica* egg DNA provides no evidence for horizontal gene transfer into the germ line of this kleptoplastic mollusc. Mol. Biol. Evol. 30, 1843–1852. 10.1093/molbev/mst084 23645554 PMC3708498

[B6] Biasini M. Bienert S. Waterhouse A. Arnold K. Studer G. Schmidt T.. (2014). SWISS-MODEL: modelling protein tertiary and quaternary structure using evolutionary information. Nucleic Acids Res. 42, W252–W258. 10.1093/nar/gku340 24782522 PMC4086089

[B7] Birkeland C. (ed.). (1997). Life and Death of Coral Reefs. New York, NY: Chapman and Hall.

[B8] Bishai W. R. Howard N. S. Winkelstein J. A. Smith H. O. (1994). Characterization and virulence analysis of catalase mutants of *Haemophilus influenzae*. Infect. Immun. 62, 4855–4860. 7927766 10.1128/iai.62.11.4855-4860.1994 PMC303198

[B9] Black D. S. Marie-Cardine A. Schraven B. Bliska J. B. (2000). The *Yersinia* tyrosine phosphatase YopH targets a novel adhesion-regulated signalling complex in macrophages. Cell. Microbiol. 2, 401–414. 10.1046/j.1462-5822.2000.00061.x 11207596

[B10] Bourne D. G. Dennis P. G. Uthicke S. Soo R. M. Tyson G. W. Webster N. (2013). Coral reef invertebrate microbiomes correlate with the presence of photosymbionts. ISME J. 7, 1452–1458. 10.1038/ismej.2012.172 23303372 PMC3695284

[B11] Butler J. MacCallum I. Kleber M. Shlyakhter I. A. Belmonte M. K. Lander E. S.. (2008). ALLPATHS: *de novo* assembly of whole-genome shotgun microreads. Genome Res. 18, 810–820. 10.1101/gr.7337908 18340039 PMC2336810

[B12] Canova M. J. Molle V. (2014). Bacterial Serine/Threonine Protein Kinases in Host-Pathogen Interactions. J. Biol. Chem. 289, 9473–9479. 10.1074/jbc.R113.529917 24554701 PMC3974997

[B13] Carver T. Harris S. R. Berriman M. Parkhill J. McQuillan J. A. (2011). Artemis: an integrated platform for visualization and analysis of high-throughput sequence-based experimental data. Bioinformatics 28, 464–469. 10.1093/bioinformatics/btr703 22199388 PMC3278759

[B14] Chaudhari A. Mahfouz M. Fialho A. M. Yamada T. Granja A. T. Zhu Y.. (2007). Cupredoxin-cancer interrelationship: azurin binding with EphB2, interference in EphB2 tyrosine phosphorylation, and inhibition of cancer growth. Biochemistry 46, 1799–1810. 10.1021/bi061661x 17249693

[B15] Chauhan J. S. Rao A. Raghava G. P. S. (2013). *In silico* platform for prediction of N-, O- and C-glycosites in eukaryotic protein sequences. PLoS ONE 8:e67008. 10.1371/journal.pone.0067008 23840574 PMC3695939

[B16] Chiang Y. R. Fang J. Y. Ismail W. Wang P. H. (2010). Initial steps in anoxic testosterone degradation by *Steroidobacter denitrificans*. Microbiology 156, 2253–2259. 10.1099/mic.0.037788-0 20413554

[B17] Coulter A. W. Talalay P. (1968). Studies on the microbiological degradation of steroid ring A. J. Biol. Chem. 243, 3238–3247. 5656367

[B18] Davy S. K. Allemand D. Weis V. M. (2012). Cell biology of cnidarian-dinoflagellate symbiosis. Microbiol. Mol. Biol. Rev. 76, 229–261. 10.1128/MMBR.05014-11 22688813 PMC3372257

[B19] De Luca G. Ventura I. Sanghez V. Russo M. T. Ajmone-Cat M. A. Cacci E.. (2013). Prolonged lifespan with enhanced exploratory behavior in mice overexpressing the oxidized nucleoside triphosphatase hMTH1. Aging Cell 12, 695–705. 10.1111/acel.12094 23648059

[B20] De Rienzo F. Gabdoulline R. R. Wade R. C. Sola M. Menziani M. C. (2004). Computational approaches to structural and functional analysis of plastocyanin and other blue copper proteins. Cell. Mol. Life Sci. 61, 1123–1142. 10.1007/s00018-004-3181-5 15141299 PMC11138940

[B21] Finn R. D. Bateman A. Clements J. Coggill P. Eberhardt R. Y. Eddy S. R.. (2014). Pfam: the protein families database. Nucleic Acids Res. 42, D222–D230. 10.1093/nar/gkt1223 24288371 PMC3965110

[B22] Galperin M. Y. Makarova K. S. Wolf Y. I. Koonin E. V (2015). Expanded microbial genome coverage and improved protein family annotation in the COG database. Nucleic Acids Res. 43, D261–D269. 10.1093/nar/gku1223 25428365 PMC4383993

[B23] Glick B. R. (2012). Plant growth-promoting bacteria: mechanisms and applications. Scientifica (Cairo). 2012, 1–15. 10.6064/2012/963401 24278762 PMC3820493

[B24] Grissa I. Vergnaud G. Pourcel C. (2007). CRISPRFinder: a web tool to identify clustered regularly interspaced short palindromic repeats. Nucleic Acids Res. 35, W52–W57. 10.1093/nar/gkm360 17537822 PMC1933234

[B25] Grosdent N. Maridonneau-Parini I. Sory M. P. Cornelis G. R. (2002). Role of Yops and adhesins in resistance of *Yersinia enterocolitica* to phagocytosis. Infect. Immun. 70, 4165–4176. 10.1128/IAI.70.8.4165-4176.2002 12117925 PMC128122

[B26] Guillard R. R. Ryther J. H. (1962). Studies of marine planktonic diatoms. I. *Cyclotella nana* Hustedt, and *Detonula confervacea* (cleve) Gran. Can. J. Microbiol. 8, 229–239. 13902807 10.1139/m62-029

[B27] Han C. Sikorski J. Lapidus A. Nolan M. Glavina Del Rio T. Tice H.. (2009). Complete genome sequence of *Kangiella koreensis* type strain (SW-125). Stand. Genomic Sci. 1, 226–233. 10.4056/sigs.36635 21304661 PMC3035244

[B28] Hemmrich G. Bosch T. C. G. (2008). Compagen, a comparative genomics platform for early branching metazoan animals, reveals early origins of genes regulating stem-cell differentiation. Bioessays 30, 1010–1018. 10.1002/bies.20813 18800383

[B29] Horinouchi M. Hayashi T. Kudo T. (2012). Steroid degradation in *Comamonas testosteroni*. J. Steroid Biochem. Mol. Biol. 129, 4–14. 10.1016/j.jsbmb.2010.10.008 21056662

[B30] Hutagalung A. H. Novick P. J. (2011). Role of Rab GTPases in membrane traffic and cell physiology. Physiol. Rev. 91, 119–149. 10.1152/physrev.00059.2009 21248164 PMC3710122

[B31] Hyatt D. Chen G.-L. Locascio P. F. Land M. L. Larimer F. W. Hauser L. J. (2010). Prodigal: prokaryotic gene recognition and translation initiation site identification. BMC Bioinformatics 11:119. 10.1186/1471-2105-11-119 20211023 PMC2848648

[B32] Hyun D. W. Shin N. R. Kim M. S. Oh S. J. Kim P. S. Whon T. W.. (2014). Endozoicomonas atrinae sp. nov., isolated from the intestine of a comb pen shell Atrina pectinata. Int. J. Syst. Evol. Microbiol. 64, 2312–2318. 10.1099/ijs.0.060780-0 24733175

[B33] Ichikawa J. Tsuchimoto D. Oka S. Ohno M. Furuichi M. Sakumi K.. (2008). Oxidation of mitochondrial deoxynucleotide pools by exposure to sodium nitroprusside induces cell death. DNA Repair (Amst.) 7, 418–430. 10.1016/j.dnarep.2007.11.007 18155646

[B34] Jeong H. Yim J. H. Lee C. Choi S.-H. Park Y. K. Yoon S. H.. (2005). Genomic blueprint of *Hahella chejuensis*jeong, a marine microbe producing an algicidal agent. Nucleic Acids Res. 33, 7066–7073. 10.1093/nar/gki1016 16352867 PMC1312362

[B35] Joshi A. D. Swanson M. S. (2011). Secrets of a successful pathogen: *Legionella* resistance to progression along the autophagic pathway. Front. Microbiol. 2:138. 10.3389/fmicb.2011.00138 21743811 PMC3127087

[B36] Juris S. J. Rudolph A. E. Huddler D. Orth K. Dixon J. E. (2000). A distinctive role for the *Yersinia* protein kinase: actin binding, kinase activation, and cytoskeleton disruption. Proc. Natl. Acad. Sci. U.S.A. 97, 9431–9436. 10.1073/pnas.170281997 10920208 PMC16881

[B37] Käll L. Krogh A. Sonnhammer E. L. L. (2007). Advantages of combined transmembrane topology and signal peptide prediction-the Phobius web server. Nucleic Acids Res. 35, 429–432. 10.1093/nar/gkm256 PMC1933244 17483518

[B38] Kanehisa M. Goto S. (2000). KEGG: kyoto encyclopedia of genes and genomes. Nucleic Acids Res. 28, 27–30. 10.1093/nar/28.1.27 10592173 PMC102409

[B39] Karp P. D. Riley M. Saier M. Paulsen I. T. Paley S. M. Pellegrini-Toole A. (2000). The EcoCyc and MetaCyc databases. Nucleic Acids Res. 28, 56–59. 10.1093/nar/28.1.56 10592180 PMC102475

[B40] Katharios P. Seth-Smith H. M. B. Fehr A. Mateos J. M. Qi W. Richter D.. (2015). Environmental marine pathogen isolation using mesocosm culture of sharpsnout seabream: striking genomic and morphological features of novel *Endozoicomonas* sp. Sci. Rep. 5:17609. 10.1038/srep17609 26639610 PMC4671022

[B41] Kenkel C. D. Meyer E. Matz M. V. (2013). Gene expression under chronic heat stress in populations of the mustard hill coral (*Porites astreoides*) from different thermal environments. Mol. Ecol. 22, 4322–4334. 10.1111/mec.12390 23899402

[B42] Kim Y. H. Lee Y. Kim S. Yeom J. Yeom S. Kim B. S.. (2006). The role of periplasmic antioxidant enzymes (superoxide dismutase and thiol peroxidase) of the Shiga toxin-producing *Escherichia coli* O157:H7 in the formation of biofilms. Proteomics 6, 6181–6193. 10.1002/pmic.200600320 17133368

[B43] Kimes N. E. Grim C. J. Johnson W. R. Hasan N. A. Tall B. D. Kothary M. H.. (2012). Temperature regulation of virulence factors in the pathogen *Vibrio coralliilyticus*. ISME J. 6, 835–846. 10.1038/ismej.2011.154 22158392 PMC3309362

[B44] Kondrashov F. A. Koonin E. V. Morgunov I. G. Finogenova T. V. Kondrashova M. N. (2006). Evolution of glyoxylate cycle enzymes in Metazoa: evidence of multiple horizontal transfer events and pseudogene formation. Biol. Direct 1:31. 10.1186/1745-6150-1-31 17059607 PMC1630690

[B45] Kosugi S. Hirakawa H. Tabata S. (2015). GMcloser: closing gaps in assemblies accurately with a likelihood-based selection of contig or long-read alignments. Bioinformatics 31, 3733–3741. 10.1093/bioinformatics/btv465 26261222

[B46] Krendel M. Mooseker M. S. (2005). Myosins: tails (and heads) of functional diversity. Physiology (Bethesda). 20, 239–251. 10.1152/physiol.00014.2005 16024512

[B47] Krzywinski M. Schein J. Birol I. Connors J. Gascoyne R. Horsman D.. (2009). Circos: an information aesthetic for comparative genomics. Genome Res. 19, 1639–1645. 10.1101/gr.092759.109 19541911 PMC2752132

[B48] Kullander K. Klein R. (2002). Mechanisms and functions of eph and ephrin signalling. Nat. Rev. Mol. Cell Biol. 3, 475–486. 10.1038/nrm856 12094214

[B49] Kurahashi M. Yokota A. (2007). *Endozoicomonas elysicola* gen. nov., sp. nov., a γ-proteobacterium isolated from the sea slug Elysia ornata. Syst. Appl. Microbiol. 30, 202–206. 10.1016/j.syapm.2006.07.003 16904280

[B50] Lai Q. Li W. Shao Z. (2012). Complete genome sequence of *Alcanivorax dieselolei* type strain B5. J. Bacteriol. 194, 6674. 10.1128/JB.01813-12 23144414 PMC3497491

[B51] Lai Q. Shao Z. (2012). Genome sequence of the alkane-degrading bacterium *Alcanivorax hongdengensis* type strain A-11-3. J. Bacteriol. 194, 6972. 10.1128/JB.01849-12 23209226 PMC3510608

[B52] Lang T. Hansson G. C. Samuelsson T. (2007). Gel-forming mucins appeared early in metazoan evolution. Proc. Natl. Acad. Sci. U.S.A. 104, 16209–16214. 10.1073/pnas.0705984104 17911254 PMC2042186

[B53] Lowe T. M. Eddy S. R. (1997). tRNAscan-SE: a program for improved detection of transfer RNA genes in genomic sequence. Nucleic Acids Res. 25, 955–964. 10.1093/nar/25.5.0955 9023104 PMC146525

[B54] Lupas A. Van Dyke M. Stock J. (1991). Predicting coiled coils from protein sequences. Science 252, 1162–1164. 10.1126/science.252.5009.1162 2031185

[B55] Marchler-Bauer A. Lu S. Anderson J. B. Chitsaz F. Derbyshire M. K. DeWeese-Scott C.. (2011). CDD: a conserved domain database for the functional annotation of proteins. Nucleic Acids Res. 39, D225–D229. 10.1093/nar/gkq1189 21109532 PMC3013737

[B56] McCutcheon J. P. Moran N. A. (2011). Extreme genome reduction in symbiotic bacteria. Nat. Rev. Microbiol. 10, 13–26. 10.1038/nrmicro2670 22064560

[B57] McGuckin M. A. Lindén S. K. Sutton P. Florin T. H. (2011). Mucin dynamics and enteric pathogens. Nat. Rev. Microbiol. 9, 265–278. 10.1038/nrmicro2538 21407243

[B58] Meikle P. Richards G. N. Yellowlees D. (1987). Structural determination of the oligosaccharide side chains from a glycoprotein isolated from the mucus of the coral *Acropora formosa*. J. Biol. Chem. 262, 16941–16947. 2890643

[B59] Mendoza M. Güiza L. Martinez X. Caraballo X. Rojas J. Aranguren L. F.. (2013). A novel agent (*Endozoicomonas elysicola*) responsible for epitheliocystis in cobia *Rachycentrum canadum* larvae. Dis. Aquat. Organ. 106, 31–37. 10.3354/dao02636 24062550

[B60] Meyer E. Aglyamova G. V. Wang S. Buchanan-Carter J. Abrego D. Colbourne J. K.. (2009). Sequencing and *de novo* analysis of a coral larval transcriptome using 454 GSFlx. BMC Genomics 10:219. 10.1186/1471-2164-10-219 19435504 PMC2689275

[B61] Meyer J. L. Paul V. J. Teplitski M. (2014). Community shifts in the surface microbiomes of the coral *Porites astreoides* with unusual lesions. PLoS ONE 9:e100316. 10.1371/journal.pone.0100316 24937478 PMC4061089

[B62] Morrow K. M. Bourne D. G. Humphrey C. Botté E. S. Laffy P. Zaneveld J.. (2015). Natural volcanic CO2 seeps reveal future trajectories for host-microbial associations in corals and sponges. ISME J. 9, 894–908. 10.1038/ismej.2014.188 25325380 PMC4817704

[B63] Myers E. W. Sutton G. G. Delcher A. L. Dew I. M. Fasulo D. P. Flanigan M. J.. (2000). A whole-genome assembly of *Drosophila*. Science 287, 2196–2204. 10.1126/science.287.5461.2196 10731133

[B64] Nadalin F. Vezzi F. Policriti A. (2012). GapFiller: a *de novo* assembly approach to fill the gap within paired reads. BMC Bioinformatics 13(Suppl. 1):S8. 10.1186/1471-2105-13-S14-S8 23095524 PMC3439727

[B65] Nawrocki E. P. Burge S. W. Bateman A. Daub J. Eberhardt R. Y. Eddy S. R.. (2015). Rfam 12.0: updates to the RNA families database. Nucleic Acids Res. 43, D130–D137. 10.1093/nar/gku1063 25392425 PMC4383904

[B66] Neave M. J. Michell C. T. Apprill A. Voolstra C. R. (2014). Whole-genome sequences of three symbiotic endozoicomonas strains. Genome Announc. 2, e00802–e00814. 10.1128/genomeA.00802-14 25125646 PMC4132622

[B67] Nishijima M. Adachi K. Katsuta A. Shizuri Y. Yamasato K. (2013). *Endozoicomonas numazuensis* sp. nov., a gammaproteobacterium isolated from marine sponges, and emended description of the genus Endozoicomonas Kurahashi and Yokota 2007. Int. J. Syst. Evol. Microbiol. 63, 709–714. 10.1099/ijs.0.042077-0 22544802

[B68] Okonechnikov K. Golosova O. Fursov M. (2012). Unipro UGENE: a unified bioinformatics toolkit. Bioinformatics 28, 1166–1167. 10.1093/bioinformatics/bts091 22368248

[B69] Pettersen E. F. Goddard T. D. Huang C. C. Couch G. S. Greenblatt D. M. Meng E. C.. (2004). UCSF Chimera–a visualization system for exploratory research and analysis. J. Comput. Chem. 25, 1605–1612. 10.1002/jcc.20084 15264254

[B70] Pierleoni A. Martelli P. L. Fariselli P. Casadio R. (2006). BaCelLo: a balanced subcellular localization predictor. Bioinformatics 22, 408–416. 10.1093/bioinformatics/btl222 16873501

[B71] Pike R. E. Haltli B. Kerr R. G. (2013). Description of *Endozoicomonas euniceicola* sp. nov. and Endozoicomonas gorgoniicola sp. nov., bacteria isolated from the octocorals Eunicea fusca and Plexaura sp., and an emended description of the genus Endozoicomonas. Int. J. Syst. Evol. Microbiol. 63, 4294–4302. 10.1099/ijs.0.051490-0 23832969

[B72] Pitulescu M. E. Adams R. H. (2010). Eph/ephrin molecules - a hub for signaling and endocytosis. Genes Dev. 24, 2480–2492. 10.1101/gad.1973910 21078817 PMC2975924

[B73] Polato N. R. Altman N. S. Baums I. B. (2013). Variation in the transcriptional response of threatened coral larvae to elevated temperatures. Mol. Ecol. 22, 1366–1382. 10.1111/mec.12163 23331636

[B74] Preston G. M. (2007). Metropolitan microbes: type III secretion in multihost symbionts. Cell Host Microbe 2, 291–294. 10.1016/j.chom.2007.10.004 18005750

[B75] Pundhir S. Kumar A. (2011). SSPred: A prediction server based on SVM for the identification and classification of proteins involved in bacterial secretion systems. Bioinformation 6, 380–382. 10.6026/97320630006380 21904425 PMC3163916

[B76] Raina J.-B. Tapiolas D. Willis B. L. Bourne D. G. (2009). Coral-associated bacteria and their role in the biogeochemical cycling of sulfur. Appl. Environ. Microbiol. 75, 3492–3501. 10.1128/AEM.02567-08 19346350 PMC2687302

[B77] Rocha E. R. Selby T. Coleman J. P. Smith C. J. Rocha E. R. Selby T.. (1996). Oxidative stress response in an Anaerobe, *Bacteroides fragilis*: a role for catalase in protection against Hydrogen Peroxide. J. Bacteriol. 178, 6895–6903. 8955312 10.1128/jb.178.23.6895-6903.1996 PMC178591

[B78] Roder C. Bayer T. Aranda M. Kruse M. Voolstra C. R. (2015). Microbiome structure of the fungid coral *Ctenactis echinata* aligns with environmental differences. Mol. Ecol. 24, 3501–3511. 10.1111/mec.13251 26018191 PMC4736464

[B79] Roterman Y. R. Benayahu Y. Reshef L. Gophna U. (2015). The gill microbiota of invasive and indigenous *Spondylus* oysters from the Mediterranean Sea and northern Red Sea. Environ. Microbiol. Rep. 7, 860–867. 10.1111/1758-2229.12315 26111733

[B80] Rua C. P. J. Trindade-Silva A. E. Appolinario L. R. Venas T. M. Garcia G. D. Carvalho L. S.. (2014). Diversity and antimicrobial potential of culturable heterotrophic bacteria associated with the endemic marine sponge *Arenosclera brasiliensis*. PeerJ 2:e419. 10.7717/peerj.419 25024903 PMC4081303

[B81] Salvioli A. Ghignone S. Novero M. Navazio L. Venice F. Bagnaresi P.. (2015). Symbiosis with an endobacterium increases the fitness of a mycorrhizal fungus, raising its bioenergetic potential. ISME J. 10, 130–144. 10.1038/ismej.2015.91 26046255 PMC4681866

[B82] Schoebel S. Cichy A. L. Goody R. S. Itzen A. (2011). Protein LidA from *Legionella* is a Rab GTPase supereffector. Proc. Natl. Acad. Sci. U.S.A. 108, 17945–17950. 10.1073/pnas.1113133108 22011575 PMC3207706

[B83] Schneiker S. Martins dos Santos V. A. P. Bartels D. Bekel T. Brecht M. Buhrmester J.. (2006). Genome sequence of the ubiquitous hydrocarbon-degrading marine bacterium *Alcanivorax borkumensis*. Nat. Biotechnol. 24, 997–1004. 10.1038/nbt1232 16878126 PMC7416663

[B84] Schwibbert K. Marin-Sanguino A. Bagyan I. Heidrich G. Lentzen G. Seitz H. (2011). A blueprint of ectoine metabolism from the genome of the industrial producer *Halomonas elongata* DSM 2581^T^. Environ. Microbiol. 13, 1973–1994. 10.1111/j.1462-2920.2010.02336.x 20849449 PMC3187862

[B85] Shinzato C. Shoguchi E. Kawashima T. Hamada M. Hisata K. Tanaka M.. (2011). Using the *Acropora digitifera* genome to understand coral responses to environmental change. Nature 476, 320–323. 10.1038/nature10249 21785439

[B86] Suzuki T. Yano K. Sugimoto S. Kitajima K. Lennarz W. J. Inoue S.. (2002). Endo-β-*N*-acetylglucosaminidase, an enzyme involved in processing of free oligosaccharides in the cytosol. Proc. Natl. Acad. Sci. U.S.A. 99, 9691–9696. 10.1073/pnas.152333599 12114544 PMC124980

[B87] Tischer S. Reineck M. Söding J. Münder S. Böttger A. (2013). Eph receptors and ephrin class B ligands are expressed at tissue boundaries in *Hydra vulgaris*. Int. J. Dev. Biol. 57, 759–765. 10.1387/ijdb.130158ab 24307295

[B88] Toth J. Cutforth T. Gelinas A. D. Bethoney K. A. Bard J. Harrison C. J. (2001). Crystal structure of an ephrin ectodomain. Dev. Cell 1, 83–92. 10.1016/S1534-5807(01)00002-8 11703926

[B89] Tout J. Siboni N. Messer L. F. Garren M. Stocker R. Webster N. S.. (2015). Increased seawater temperature increases the abundance and alters the structure of natural Vibrio populations associated with the coral *Pocillopora damicornis*. Front. Microbiol. 6:432. 10.3389/fmicb.2015.00432 26042096 PMC4435422

[B90] Trench R. K. (1979). The cell biology of plant-animal symbiosis. Annu. Rev. Plant Physiol. 30, 485–531. 10.1146/annurev.pp.30.060179.002413

[B91] Twan W. H. Hwang J. S. Lee Y. H. Wu H. F. Tung Y. H. Chang C. F. (2006). Hormones and reproduction in scleractinian corals. Comp. Biochem. Physiol. A Mol. Integr. Physiol. 144, 247–253. 10.1016/j.cbpa.2006.01.011 16488637

[B92] Vezzulli L. Pezzati E. Huete-Stauffer C. Pruzzo C. Cerrano C. (2013). 16SrDNA Pyrosequencing of the Mediterranean Gorgonian Reveals a link among alterations in Bacterial Holobiont Members, Anthropogenic Influence and disease outbreaks. PLoS ONE 8:e67745. 10.1371/journal.pone.0067745 23840768 PMC3694090

[B93] Visick K. L. Ruby E. G. (1998). The periplasmic, group III catalase of *Vibrio fischeri* is required for normal symbiotic competence and is induced both by oxidative stress and by approach to stationary phase. J. Bacteriol. 180, 2087–2092. 9555890 10.1128/jb.180.8.2087-2092.1998 PMC107134

[B94] Wang D. Zetterstrom C. E. Gabrielsen M. Beckham K. S. H. Tree J. J. Macdonald S. E.. (2011). Identification of Bacterial target proteins for the Salicylidene Acylhydrazide class of virulence-blocking compounds. J. Biol. Chem. 286, 29922–29931. 10.1074/jbc.M111.233858 21724850 PMC3191033

[B95] Whitmore S. E. Lamont R. J. (2012). Tyrosine phosphorylation and bacterial virulence. Int. J. Oral Sci. 4, 1–6. 10.1038/ijos.2012.6 22388693 PMC3412661

[B96] Wilson K. (2001). Preparation of genomic DNA from bacteria. Curr. Protoc. Mol. Biol. Chapter 2; Unit 2.4. 10.1002/0471142727.mb0204s56 18265184

[B97] Wu H. J. Seib K. L. Edwards J. L. Apicella M. A. McEwan A. G. Jennings M. P. (2005). Azurin of pathogenic *Neisseria* spp. is involved in defense against hydrogen peroxide and survival within cervical epithelial cells. Infect. Immun. 73, 8444–8448. 10.1128/IAI.73.12.8444-8448.2005 16299348 PMC1307039

[B98] Yamada T. Fialho A. M. Punj V. Bratescu L. Gupta T. K. Chakrabarty A. M. (2005). Internalization of bacterial redox protein azurin in mammalian cells: entry domain and specificity. Cell. Microbiol. 7, 1418–1431. 10.1111/j.1462-5822.2005.00567.x 16153242

[B99] Yang C. S. Chen M. H. Arun A. B. Chen C. A. Wang J. T. Chen W. M. (2010). *Endozoicomonas montiporae* sp. nov., isolated from the encrusting pore coral *Montipora aequituberculata*. Int. J. Syst. Evol. Microbiol. 60, 1158–1162. 10.1099/ijs.0.014357-0 19666790

[B100] Yin J. Li L. Shaw N. Li Y. Song J. K. Zhang W.. (2009). Structural basis and catalytic mechanism for the dual functional endo-&beta;-N-acetylglucosaminidase A. PLoS ONE 4:e4658. 10.1371/journal.pone.0004658 19252736 PMC2646837

[B101] Zhou Y. Liang Y. Lynch K. H. Dennis J. J. Wishart D. S. (2011). PHAST: a fast phage search tool. Nucleic Acids Res. 39, W347–W352. 10.1093/nar/gkr485 21672955 PMC3125810

