# Supplementary material for: Genomic Insight into the Host–Endosymbiont Relationship of Endozoicomonas montiporae CL-33T with its Coral Host
Source: Front Microbiol. 2016 Mar 8;7:251. doi: 10.3389/fmicb.2016.00251 (PMC4781883; doi:10.3389/fmicb.2016.00251)
Supplement: Supplementary file 5 [file Image1.PDF]

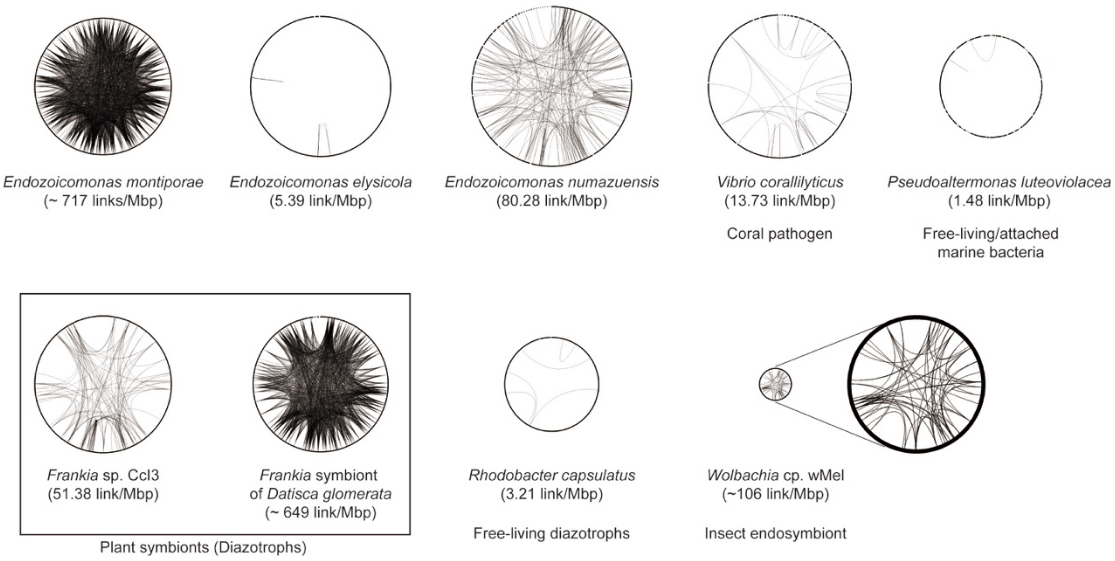

Supplementary Figure S1. Repeat densities of various bacterial genomes. Repeat sequences were connected if the sequences shared >98% of identities (see Materials and Methods). For incomplete genomes, scaffolds were sorted by their length and draw sequentially. Sizes of circular plots were normalized by corresponding genome size of each bacterial genome.
