# Supplementary material for: Genomic Insight into the Host–Endosymbiont Relationship of Endozoicomonas montiporae CL-33T with its Coral Host
Source: Front Microbiol. 2016 Mar 8;7:251. doi: 10.3389/fmicb.2016.00251 (PMC4781883; doi:10.3389/fmicb.2016.00251)
Supplement: Supplementary file 6 [file Image2.PDF]

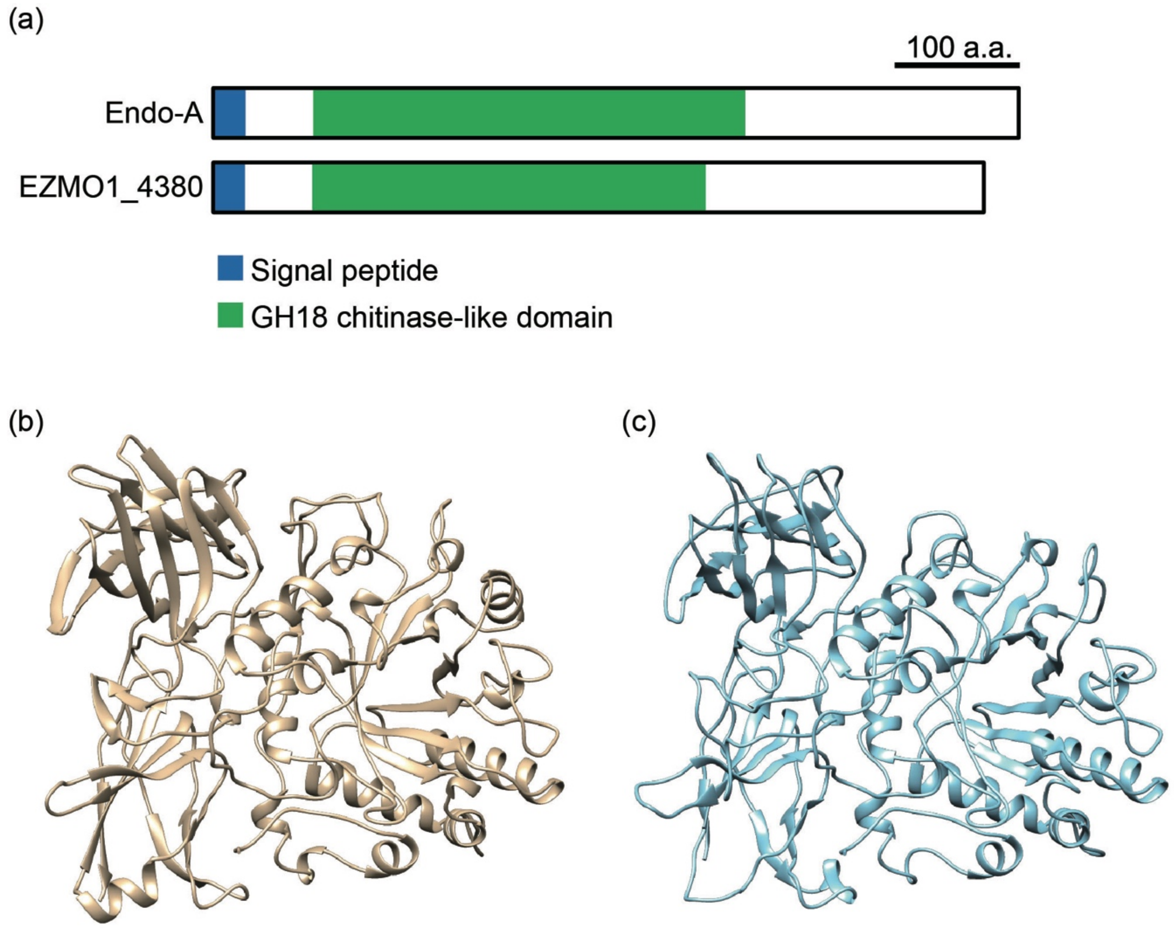

Supplementary Figure S2. The domain architectural (a) and 3D structural comparison of the endo-β-*N*-acetylglucosaminidase A from *Arthrobacter protophormiae* (b) and EZMO1_4380 (c).
