# Supplementary material for: Genomic Insight into the Host–Endosymbiont Relationship of Endozoicomonas montiporae CL-33T with its Coral Host
Source: Front Microbiol. 2016 Mar 8;7:251. doi: 10.3389/fmicb.2016.00251 (PMC4781883; doi:10.3389/fmicb.2016.00251)
Supplement: Supplementary file 7 [file Image3.PDF]

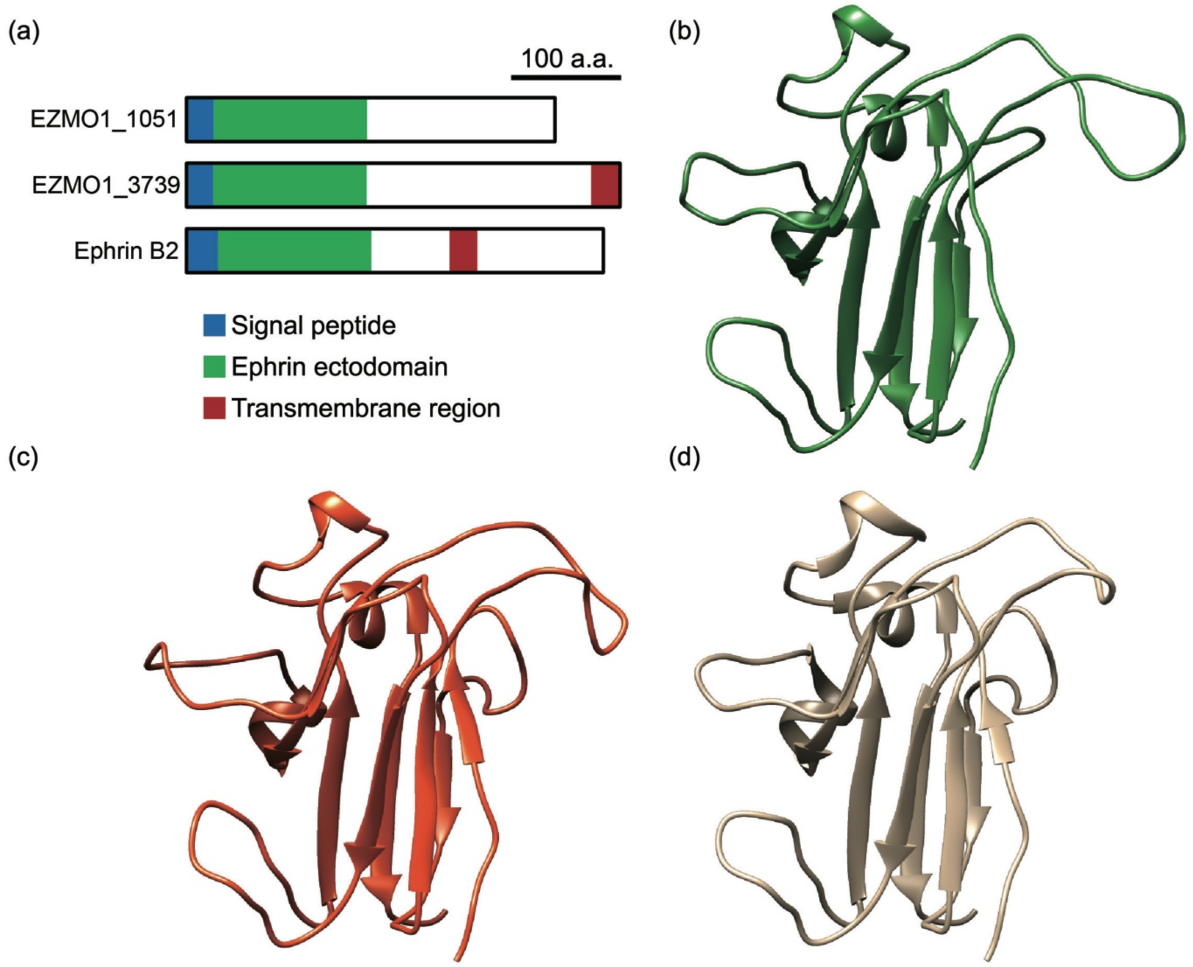

Supplementary Figure S3. Structure analysis of ephrin ligands. The protein domain structure of *E. montiporae* proteins were compared with the mouse ephrin-B2 (a). The highly conserved ephrin ectodomains from *E. montiporae* were further analyzed with homology modeling and the predicted structures (b and c) were highly similar to mouse ephrin-B2 (d).
