# Supplementary material for: Genomic Insight into the Host–Endosymbiont Relationship of Endozoicomonas montiporae CL-33T with its Coral Host
Source: Front Microbiol. 2016 Mar 8;7:251. doi: 10.3389/fmicb.2016.00251 (PMC4781883; doi:10.3389/fmicb.2016.00251)
Supplement: Supplementary file 8 [file Image4.PDF]

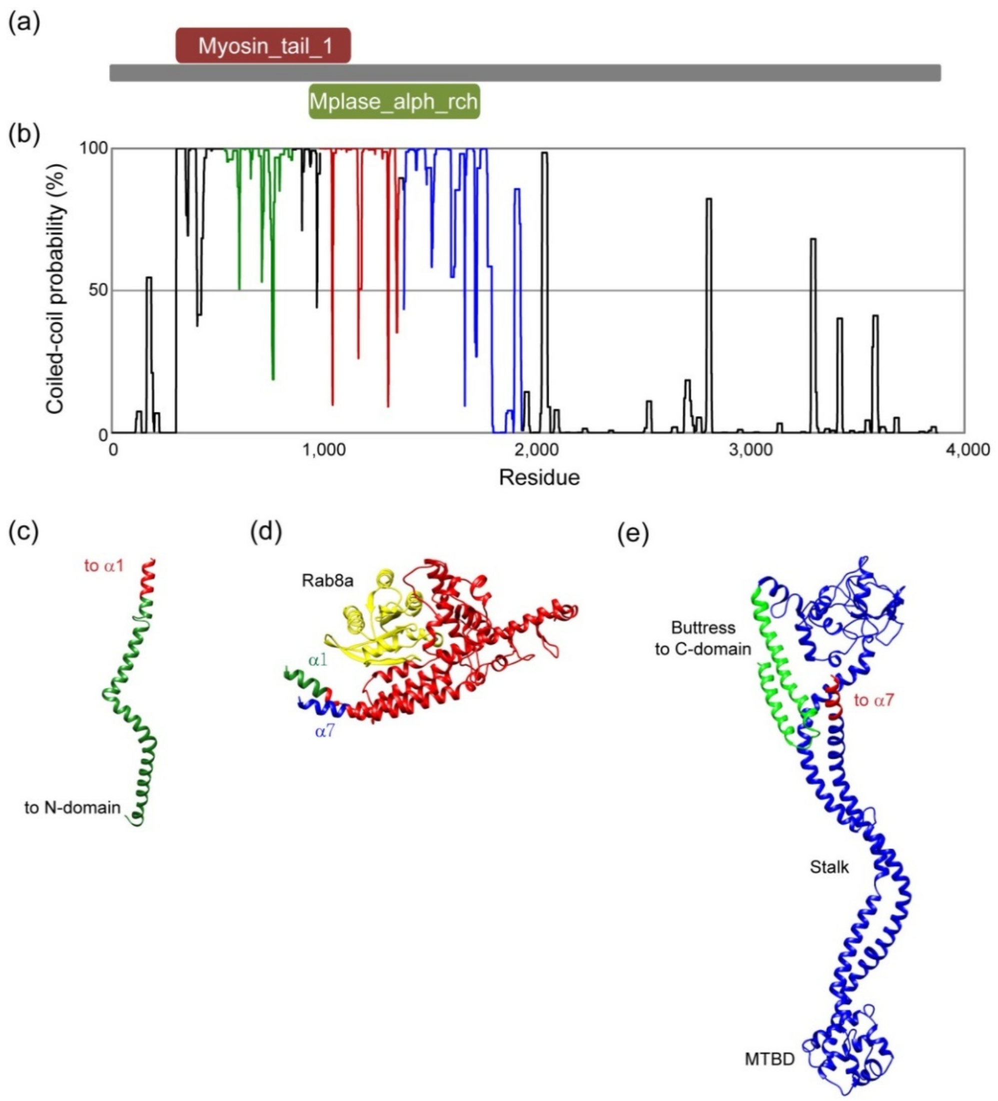

Supplementary Figure S4. Structure analysis of EZMO1_3398. The protein domains, Myosin_tail_1 and Mplase_alph_rch, were located at the N-terminal region (a), which was overlapped with the coiled-coil structure region in EZMO1_3398 (b). The structure prediction showed EZMO1_3398 contained three known substructures, myosin tail (c), LidA (d) and dynein stalk with mircotube binding domain (MTBD) as well as the buttress (e). The connections of the three substructures were indicated by different color. The human Rab8a was superimposed to the central LidA domain according the model template 3TNF.
