# Supplementary material for: Genomic Insight into the Host–Endosymbiont Relationship of Endozoicomonas montiporae CL-33T with its Coral Host
Source: Front Microbiol. 2016 Mar 8;7:251. doi: 10.3389/fmicb.2016.00251 (PMC4781883; doi:10.3389/fmicb.2016.00251)
Supplement: Supplementary file 9 [file Image5.PDF]

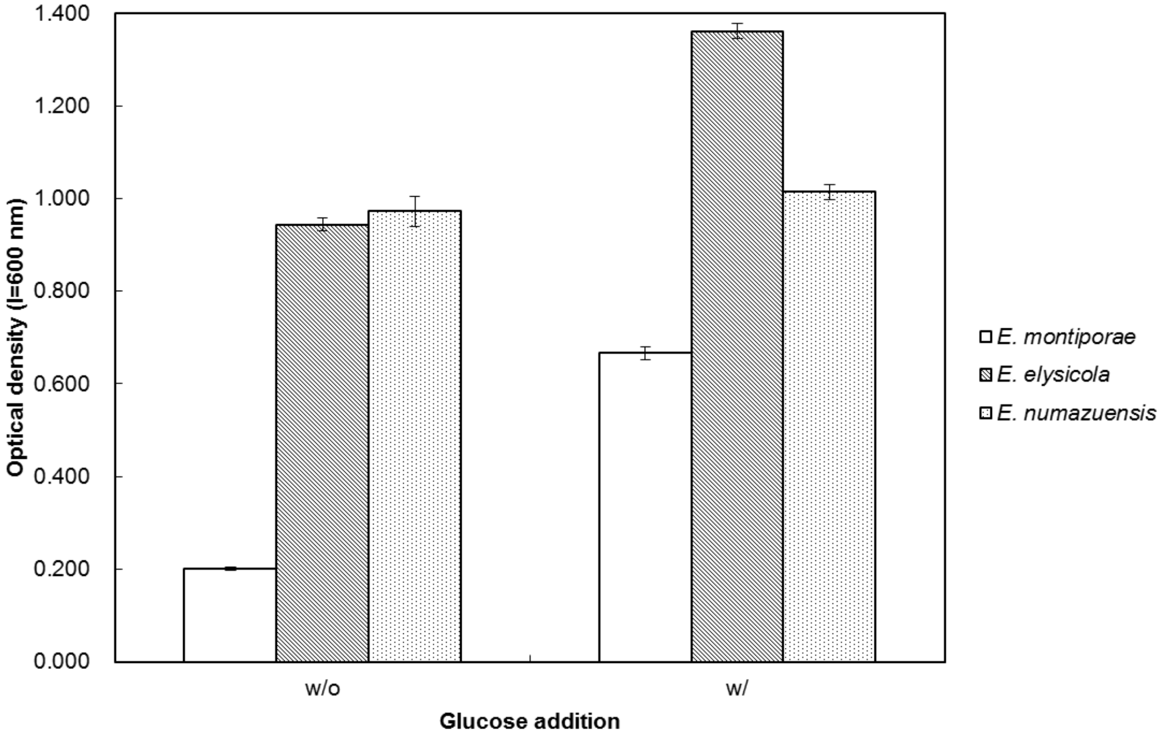

Supplementary Figure S5. Maximum cell densities of three *Endozoicomonas* species when cultured in MMB, with (w/) or without (w/o) glucose (0.1 %, w/v). Error bar = SD (estimated from triplicate tests).
